# Crystallographic and NMR Investigation of Ergometrine and Methylergometrine, Two Alkaloids from *Claviceps Purpurea*

**DOI:** 10.3390/molecules25020331

**Published:** 2020-01-14

**Authors:** Fiorella Meneghetti, Patrizia Ferraboschi, Paride Grisenti, Shahrzad Reza Elahi, Matteo Mori, Samuele Ciceri

**Affiliations:** 1 Department of Pharmaceutical Sciences, University of Milan, via L. Mangiagalli, 25, 20133 Milano, Italy; fiorella.meneghetti@unimi.it (F.M.); matteo.mori@unimi.it (M.M.); 2 Department of Medical Biotechnology and Translational Medicine, University of Milan, Via Saldini 50, 20133 Milano, Italy; patrizia.ferraboschi@unimi.it (P.F.); shahrzad.rezaelahi@gmail.com (S.R.E.); 3 Chemical-Pharmaceutical Consulting and IP Management, Viale Giovanni da Cermenate 58, 20141 Milano, Italy; grisenti.paride60@gmail.com

**Keywords:** ergot, 9,10-unsaturated ergoline, alkaloids, ^15^N NMR, ^13^C NMR, ^1^H NMR, X-ray analysis, oxytocic activity

## Abstract

Ergometrine and methylergometrine are two alkaloids that are used as maleate salts for the prevention and control of postpartum hemorrhage. Although the two molecules have been known for a long time, few and discordant crystallographic and NMR spectroscopic data are available in the literature. With the aim of providing more conclusive data, we performed a careful NMR study for the complete assignment of the ^1^H, ^13^C, and ^15^N NMR signals of ergometrine, methylergometrine, and their maleate salts. This information allowed for a better definition of their conformational equilibria. In addition, the stereochemistry and the intermolecular interactions in the solid state of the two maleate salts were deeply investigated by means of single-crystal X-ray diffraction, showing the capability of these derivatives to act as both hydrogen-bond donors and acceptors, and evidencing a correlation between the number of intermolecular interactions and their different solubility.

## 1. Introduction

**Figure 1:**
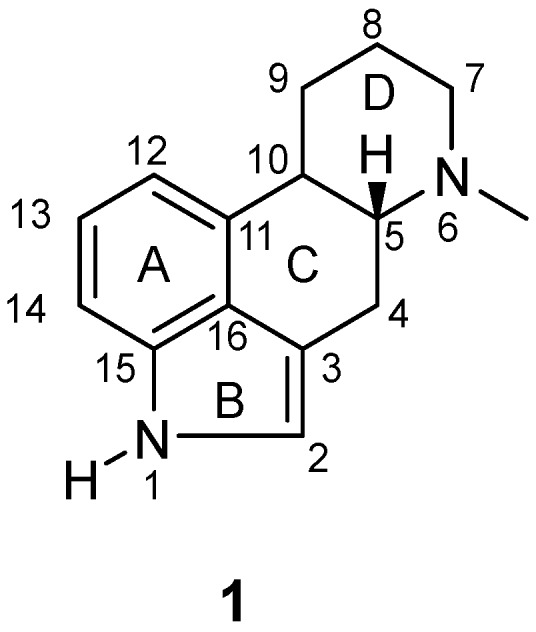
Structure of ergoline (**1**).

The fungus *Claviceps purpurea* (commonly known as ergot) is a plant pathogen that produces a family of toxic alkaloids endowed with pharmacological properties [1,2]. These ergot alkaloids are derivatives of the tetracyclic ergoline **1** (Figure 1) and can be chemically classified by the nature of the substituent at the 8-position. 

**Figure 2:**
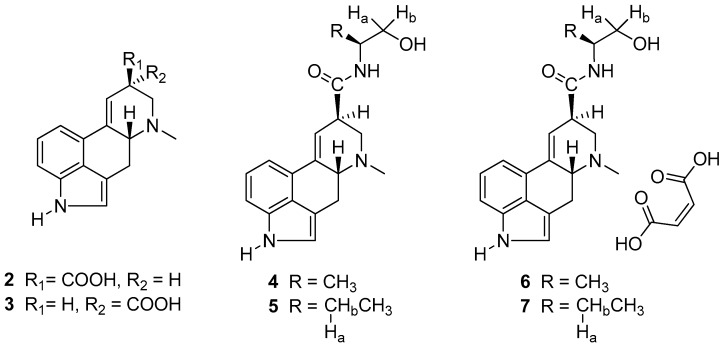
Structures of *d*-lysergic (**2**), *d*-isolysergic acid (**3**), ergometrine (**4**), methylergometrine (**5**), ergometrine maleate (**6**), and methylergometrine maleate (**7**).

From a therapeutic point of view, the *d*-lysergic acid amides are in a prominent position among the ergot alkaloids. The *d*-lysergic acid **2** carries a carboxyl group at the 8β position (*cis* with respect to the H-5) and a 9–10 double bond (Figure 2). The presence of the double bond is responsible for the easy and spontaneous isomerization of the carboxyl substituent at the C-8 stereocenter, leading to mixtures of *d*-lysergic and *d*-isolysergic acid **3**. In the tetracyclic moiety of compounds **2** and **3**, two stereocenters are present, namely at the 5 and 8 positions. The stereocenter at the 5 position presents an *R* configuration, while the substituent at the 8 position can be either in the α-configuration (*trans* with respect to the H-5) or in the β-configuration (*cis* with respect to the H-5). The α-isomer is usually indicated by the prefix iso- or by the ending –inine. The β-isomers are usually endowed with a significant biological activity.

Among the *d*-lysergic derivatives, the natural amide ergometrine (ergonovine) **4** and the semisynthetic methylergometrine **5** (methylergonovine) (Figure 2) are endowed with uterotonic activity, and for this reason, their maleate salts are administered in the third stage of labor for the prevention of postpartum hemorrhage [3,4].

Although ergometrine **4** was isolated in 1932 [5] and the first synthesis of methylergometrine **5**, starting from the lysergic azide, was published in 1943 [6], only a few spectroscopic and crystallographic studies are reported in the literature. In two 2017 Chinese patents [7,8], the ^1^H NMR data of the ergometrine **4** are reported but not assigned; in 1974 [9], the assigned ^13^C NMR (60 MHz) data were reported. In the case of ergometrine maleate, the XRD and the single-crystal X-ray diffraction [10] are reported, and only the ^1^H NMR (400 MHz) characterization is available [11]. Methylergometrine **5** was analyzed by ^1^H NMR (values not assigned) in a 2014 WO patent [12]; the corresponding maleate was characterized by XRD, IR, and DSC in a 2015 Chinese patent [13] and by synchrotron powder diffraction data [14]. These incomplete (and sometimes contradictory) NMR and crystallographic analyses prompted us to gather new and more conclusive data for the two amides, **4** and **5**. Considering the easy epimerization of these compounds, we decided to expand our investigations to their maleate salts, **6** and **7**, as well. Indeed, ergometrine **4** and methylergometrine **5** are always obtained as mixtures of epimers, while the corresponding maleate salts can be obtained as pure β-isomers by simple crystallization.

In a continuing effort to fully characterize molecules that exert important therapeutic properties [15,16,17], a complete NMR (^1^H, ^13^C, and ^15^N) characterization of compounds **4**, **5**, **6**, and **7** was carried out. Moreover, a detailed crystallographic investigation was performed on **6** and **7** in order to explore their conformational features. In addition, we carefully analyzed the hydrogen-bonding interactions of these molecules, as the earlier report did not account for their supramolecular aggregation.

## 2. Results and Discussion

Ergometrine **4** and methylergometrine **5** were obtained by treatment of the corresponding commercially available maleates, **6** and **7**, with sodium hydrogen carbonate as described in the Materials and Methods section.

### 2.1. NMR Spectroscopy

The NMR study was carried out on **4** and **5** and on their maleate salts **6** and **7** (the clinically administered derivatives). Compounds **4** and **5** were dissolved in DMSO-*d*_6_, while compounds **6** and **7** were dissolved in D_2_O. Although in both solvents the ^1^H NMR spectra of **6** and **7** present broadened signals and a slight concentration dependence of chemical shifts, D_2_O was chosen in these cases due to more resolved NMR spectra. ^1^H, ^13^C, and ^15^N resonances (Table 1, Table 2 and Table 3) were unambiguously assigned combining information from 1D and 2D NMR (COSY, NOESY, HSQC, ^1^H-^13^C HMBC, and ^1^H-^15^N HMBC) experiments. 

The proton assignments were accomplished using the general knowledge of chemical shift dispersion with the aid of the COSY, HSQC, and NOESY experiments. Starting from the characteristic resonance of H-9 (6.34, 6.35, 6.46, and 6.49 ppm for **4**, **5**, **6**, and **7,** respectively), we were able to assign the resonances of all the other protons, especially the resonance of H-8. The interpretation of the ^1^H NMR spectra of compounds **4** and **5** was further facilitated by the resonances of exchangeable protons H-1, CH_2_O*H*, and CON*H*.

**Figure 3:**
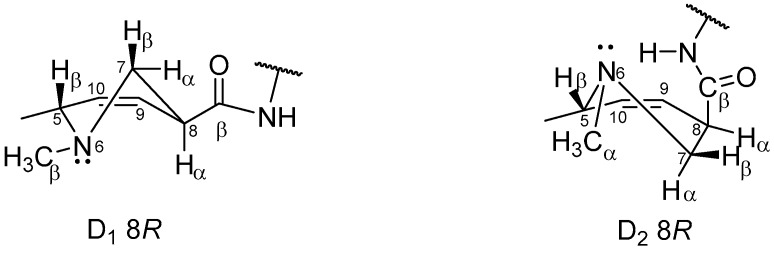
Possible conformations of ergoline D-ring in solution.

The NOESY experiments were performed to integrate structural data with stereochemical information. Despite the poorly resolved 2D spectra of NOESY experiments obtained for **6** and **7** due to the broad nature of the D-ring proton signals, some information about the stereochemistry and conformation of the D-ring was collected by observing the NOE correlation of the methyl group bound to the nitrogen atom at the 6 position. This methyl group interacts sterically with H-5β, H-7β, H-7α, and H-4β, indicating—as previously described by Kidric et al. [18] for 9,10-unsatured ergolines dissolved in solvents such as DMSO and water—that the D-ring was preferentially in its D_1_ 8*R* half-chair conformation. The very weak NOE correlation of N–CH_3_ with H-8 α and H-4 α could be explained by the existence of a conformational equilibrium in solution between D_1_ 8*R* and D_2_ 8*R* half-chair conformations (Figure 3). When the D-ring is in its D_2_ 8*R* configuration, the methyl group is in the α position, which results in a feeble NOE effect. The existence of this conformational equilibrium in aqueous solution also explains the broad nature of the C- and D-ring proton signals. 

The analysis of NOESY spectra of the free bases, **4** and **5**, gave us a clear picture of the spatial interactions of protons at the 4 position with H-5β and of protons at the 7 position with H-8α. The NOE correlations of H-5β (3.00–2.93 ppm) with one of the two protons at the 4 position (3.46 ppm) and of H-8α (3.40–3.36 ppm) with one of the two protons at the 7 position (3.00 ppm for **4** and 3.02 ppm for **5**) allowed us to unequivocally identify H-4β and H-7α protons. The absence of spatial interactions between the H-8 and H-5 protons was a clue, but not an absolute proof, of their position on opposite faces of the D-ring. When N-CH_3_ (2.44 ppm for **4**; 2.45 ppm for **5**) was irradiated, NOE enhancement was observed for H-5β, suggesting that the analyzed **4** and **5** in DMSO-d_6_ were in their D_1_ 8*R* half-chair conformation. The CH, CH_2_, and CH_3_ carbon atoms were assigned based on chemical shift analysis and confirmed by the HSQC experiment. In particular, the predicted deshielding of C-5 and C-7 was observed, both near the nitrogen atom in position 6, further confirming the right ^1^H NMR assignment of the protons bound to these carbon atoms. In the ^13^C NMR spectra of compounds **6** and **7**, the broad nature and low intensity of the C-4, C-8, N-CH_3_, C-7, C-5, and C-9 signals can be explained by the previously reported conformational equilibrium between the D_1_ 8*R* and D_2_ 8*R* half-chair conformations. The quaternary carbon atoms were unambiguously assigned using the information obtained from ^1^H-^13^C HMBC experiments (Table 4). Following this approach, the ^13^C resonance assignments of **4** were in accordance with the previously reported ones [9].

^1^H-^15^N HMBC experiments were performed in order to assign the resonances of the three chemically different nitrogen atoms in the examined compounds and allowed us to confirm their hypothesized structure. For compounds **6** and **7**, the indole nitrogen N-1 (−253.3 ppm) couples with H-2, H-14, and H-13, the amidic nitrogen (−248.3 ppm for **6**; −251.4 ppm for **7**) couples with the protons present on the amidic moiety, and curiously but not unexpectedly, N-6 (−332.8 ppm for **6**; −332.7 ppm for **7**) couples very weakly only with H-4α, which gives a well-resolved signal in the ^1^H NMR spectrum. For compounds **4** and **5**, the indole nitrogen N-1 (−251.1 ppm for **4**; −251.0 ppm for **5**) couples with H-1, H-2, H-14, and H-13, the amidic nitrogen (−255.5 ppm for **4**; −258.1 ppm for **5**) couples with the protons of the amidic moiety, and N-6 (−340.3 ppm for **4**; −340.2 ppm for **5**) couples with N-CH_3_, H-7β, and H-5.

### 2.2. X-ray Analysis

**Figure 4:**
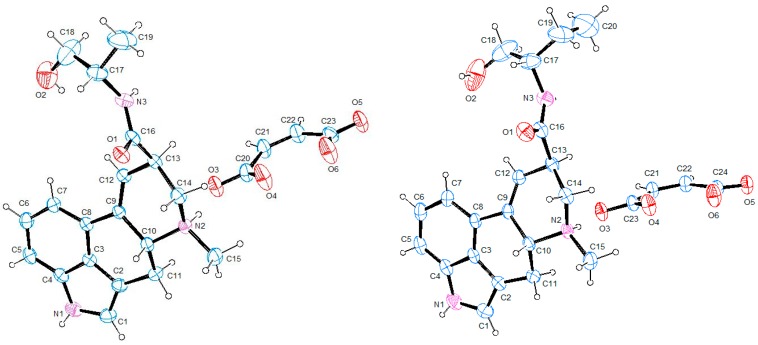
ORTEP drawings [19] of **6** (**left**) and **7** (**right**), with the arbitrary atom numbering (ellipsoids are at 40% probability and H atoms are represented as spheres of arbitrary radii).

Since the structure adopted by a given compound upon crystallization could exert a profound effect on the solid-state properties of the system, we decided to perform a crystallographic analysis of derivatives **6** and **7**. Their X-ray molecular structures are presented, as ORTEP views [19], in Figure 4.

The maleate salt of ergometrine, compound **6**, crystallized in the orthorhombic P2_1_2_1_2_1_ system, as previously reported in the literature [20], while the maleate salt of methylergometrine **7** crystallized in a different space group, namely the monoclinic P2_1_ [14].

The dianionic maleate group bridges two neighboring alkaloid molecules, whose conformation is mainly determined by a central rigid core, consisting of an indole plane connected to a six-membered ring and a tetra-hydro-pyridine. Since the absolute configuration of C5 of the starting material was known, it was possible to determine by X-ray diffraction the relative configuration of C8 in the two crystal structures. The single crystal diffraction data unambiguously confirmed that the substituent at the 8-position is equatorially oriented (*cis* with respect to H-5), and with respect to an absolute configuration R at the 5-position, this relative orientation means the *R* configuration of C8. The cores of the two molecules are closely related: the ring C of the central skeleton is in a slightly distorted envelope conformation, as indicated by the puckering parameters [21] Q_T_ (total puckering amplitude) = 0.3803(1)Å, θ (torsion angle) = 53.8(1)° for **6**, and Q_T_ = 0.3630(1)Å, θ = 51.8(1)° for **7**, whereas ring D adopts a distorted chair conformation, with puckering coordinates [21] Q_T_ = 0.5208 (1)Å, θ = 126.1(1)° for **6** and Q_T_ = 0.5170 (1)Å, θ = 127.7(1)° for **7**, which is in agreement with the previously reported structures. The conformation of the tetracyclic system is also conserved in the ergometrinine crystal structure [22], which is the biologically inactive isomer of **4**.

**Figure 5:**
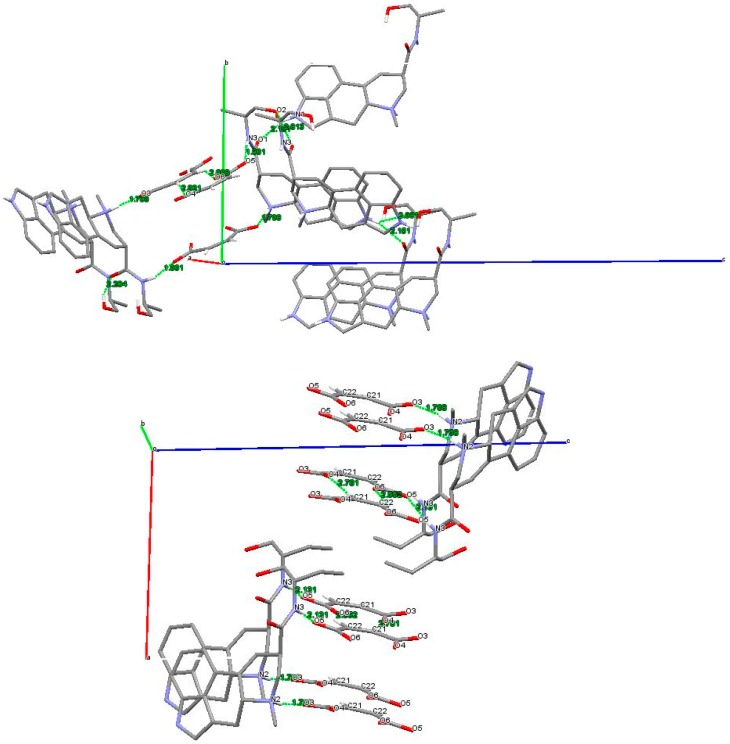
The dashed lines show the intermolecular contacts of **6** (**above**), viewed about along the *a*-axis, and of **7** (**below**)**,** viewed about along the *b*-axis (only the hydrogens involved in the interactions are shown).

The crystal lattice of both structures is dominated by hydrogen bonds (Figure 5); in **6,** in particular, the negatively charged carboxylate groups of the anions are engaged with a hydrogen belonging to the positively charged amino groups of the cations; moreover, bifurcated hydrogen bonds linking O1 with N1 and O2 of symmetry-related ergometrine molecules contribute to stabilize the crystals. In addition, Cπ-H···O contacts between neighboring anions give rise to the formation of molecular chains along the *a*-axis of the unit cell, further consolidating the crystal packing. In **7**, we revealed some differences with respect to the previously reported structure [14]: in particular, the absence of the intermolecular interaction between the amidic oxygen and the NH of ring B and the presence of Cπ-H···O contacts between neighboring anions, which give rise to the formation of molecular chains along the *b*-axis of the unit cell.

The hydrogen bond geometry of **6** and **7**, summarized in Table 5, evidenced the capacity of these derivatives to act as both hydrogen-bond donors and acceptors.

Moreover, the crystallographic analysis suggests that the lower solubility of **6** in the crystallization mixture (1:1 acetone/ethanol) is caused by the presence of an extensive system of hydrogen bonding. Therefore, the higher solubility of **7** may be correlated to the reduced number of intermolecular interactions found in the solid state, leading to a decreased crystal lattice energy. The less soluble **6** crystallizes in a compact structure as prisms, while the poor-quality crystals of **7** (obtained from a 1:1 ethanol/water crystallization mixture) are assembled in transparent platelets, showing a reasonable correlation between the different morphological aspect of the crystals and their solubility.

## 3. Materials and Methods

### 3.1. Chemistry

Ergometrine maleate and methylergometrine maleate, all reagents and solvents were purchased from Sigma-Aldrich (Merck, Kenilworth, NJ, USA).

#### 3.1.1. Ergometrine **4** from the Corresponding Maleate **6**

To a suspension of commercial ergometrine maleate (50 mg, 0.11 mmol) in ethyl acetate (10 mL), a saturated aqueous sodium bicarbonate solution (10 mL) was added. After stirring at room temperature in the dark for 30 min, the layers were separated. The aqueous phase was extracted with ethyl acetate (2 × 10 mL). The collected organic phases were dried over sodium sulfate and filtered. After solvent removal under reduced pressure, a white solid was obtained (35 mg, 0.11 mmol, 96%).

[α]_D_^20^ = −17.9° (c = 1; pyridine) lit. [6] −16° (1)

#### 3.1.2. Methylergometrine **5** from the Corresponding Maleate **7**

Methylergometrine **5** was obtained following the previously described procedure for ergometrine **4**, starting from commercial methylergometrine maleate (50 mg, 0.11 mmol). A white solid was obtained (34 mg, 0.10 mmol, 91%).

[α]_D_^20^ = −49.8° (c = 0.4; pyridine) lit. [6] −45° (2)

### 3.2. NMR Spectroscopy

NMR spectra were recorded on a Bruker AVANCE 500 spectrometer (Bruker, Billerica, MA, USA) equipped with a 5 mm broadband inverse (BBI) detection probe with field *z*-gradient operating at 500.13, 125.76, and 50.69 MHz for ^1^H, ^13^C, and ^15^N respectively. NMR spectra were recorded at 300 K for compounds **4** and **5** in DMSO-*d*_6_ (isotopic enrichment 99.9 atom % D), for their maleate salts, **6** and **7**, in D_2_O (isotopic enrichment 99.9 atom % D) solution. The data were collected and processed by XWIN-NMR software (version 3.5, Bruker, Billerica, MA, USA) running on a PC with Microsoft Windows 7. The samples (10 mg) were dissolved in the appropriate solvent (0.75 mL) in a 5 mm NMR tube. The acquisition parameters for 1D were as follows: ^1^H spectral width of 5000 Hz and 32 K data points providing a digital resolution of ca. 0.305 Hz per point, relaxation delay 10 s; ^13^C spectral width of 29,412 Hz, and 64 K data points providing a digital resolution of ca. 0.898 Hz per point, relaxation delay 2 s. The experimental error in the measured ^1^H-^1^H coupling constants was ±0.5 Hz. Chemical shifts (δ) of the ^1^H NMR and ^13^C NMR spectra are reported in ppm using the central peak of DMSO-*d*_6_ signals (2.50 ppm for ^1^H; 39.52 ppm for ^13^C) for compounds **4** and **5** and using methanol as external reference in the spectra recorded in D_2_O (signals of CH_3_OH in D_2_O: 3.34 ppm for ^1^H; 49.50 ppm for ^13^C). Methylergometrine maleate D_2_O solution showed a pH of 4.5, while ergometrine maleate D_2_O solution showed a pH of 4.2. The splitting pattern abbreviations are as follows: s, singlet; d, doublet; t, triplet; q, quartet; m, multiplet; and br, broad signal. For two-dimensional experiments, standard Bruker microprograms using gradient selection (gs) were applied. Gs-COSY-45 and phase sensitive gs-NOESY experiments were acquired with 512 t1 increments; 2048 t2 points; and a spectral width of 11.0 ppm. The gs-NOESY experiments were performed with a mixing time of 0.800 s on samples degassed under a flush of argon in a screwcap sample tube. There were not significant differences in the results obtained at different mixing times (0.5–1.5 s). The acquisition data for gs-HSQC and gs-HMBC experiments were acquired with 512 t1 increments, 2048 t2 points, and a spectral width of 11.0 ppm for ^1^H and 200 ppm for ^13^C. Delay values were optimized for ^1^*J*_C,H_ 140 Hz and ^n^*J*_C,H_ 8.0 Hz. For ^1^H-^15^N HMBC experiments, nitromethane was used as the external reference, referencing the resonance of its ^15^N at 0.00 ppm. Samples concentration: 10 mg/0.75 mL corresponding to 41, 39, 30, and 29 mM for **4**, **5**, **6**, and **7**, respectively. The acquisitions were performed setting an acquisition time of 0.5 s, a delay between scans of 10 s, a ^1^*J*_N,H_ value of 90.0 Hz, and a ^n^*J*_N,H_ value of 1.9 Hz. This last parameter was set after several attempts between 1 and 10 Hz. Total ^1^H-^15^N HMBC experimental time: 64 h.

The NMR spectra are available in the Supplementary Materials section.

### 3.3. X-ray Crystallography

Crystals of **6** were obtained as pale-yellow prisms from a 1:1 acetone/ethanol solution at room temperature. Poor-quality crystals of **7** were obtained, after many attempts, as colorless elongated platelets from a 1:1 ethanol/water solution at room temperature. All experiments were carried out in the dark to avoid degradation.

Diffraction data for the crystals of **6** and **7** were collected with a Bruker-AXS CCD-based three-circle diffractometer, working at ambient temperature with graphite-monochromatized Mo-Kα X-ray radiation (λ = 0.7107 Å).

X-ray diffraction data in the θ range 2–25° were collected acquiring three sets of 600 bidimensional CCD frames with the following operative conditions: omega rotation axis, scan width 0.3°, acquisition time 40 s, sample-to-detector distance 60 mm, and phi angle fixed at three different values (0°, 120°, 240°) for the three different sets.

Omega-rotation frames were processed with the SAINT software [23] for data reduction (including intensity integration, background, Lorentz, and polarization corrections) and for the determination of accurate unit-cell dimensions, which were obtained by least-squares refinement of the positions of 2023 independent reflections with I > 10σ(I) in the θ range 2–19°. Absorption effects were empirically evaluated by the SADABS software [24], and absorption correction was applied to the data (0.87 and 0.99 min and max transmission factor). The structures were solved by direct methods (SIR-14) [25] and completed by iterative cycles of full-matrix least squares refinement on F_0_^2^ and DF synthesis using the WingX.v2014.1 [26] program. The hydrogen atoms bonded to carbon were included at geometrically calculated positions and refined using a riding model. Uiso(H) is defined as 1.2 Ueq of the parent carbon atoms for the phenyl and methylene residues and 1.5 Ueq of the parent carbon atoms for the methyl group. Crystallographic data has been deposited with the Cambridge Crystallographic Data Centre under CCDC deposition numbers 1970319 (**6**) and 1970320 (**7**). Copies of this information may be obtained free of charge from the Director, CCDC, 12 Union Road, Cambridge CB2 1EY, UK (fax: +44-1223-336033; e-mail: deposit@ccdc.cam.ac.uk or www:http://www.ccdc.cam.ac.uk).

Crystal data for **6**: C_23_H_27_N_3_O_6_, *M*_r_ = 441.47 g/mol, Orthorhombic, Space group *P*2_1_2_1_2_1_, *a* = 5.7139(7) Å, *b* = 12.3104(1) Å, *c* = 33.2683(4) Å, *V* = 2340(1) Å^3^, *Z* = 4, *D_calc_* = 1.253 Mg/m^3^, F(000) = 936, *R* = 0.057 (reflections collected/unique = 4113/2921), *wR2* = 0.146, *T* = 294(2)K, and *GOF* = 1.026. The reflections were collected in the range 1.22° ≤ θ ≤ 25.05° (limiting indices = −6 ≤ h ≤ 6, −14 ≤ k ≤ 14, −39 ≤ l ≤ 39) employing a 0.30 × 0.20 × 0.07 mm crystal. The residual positive and negative electron densities in the final map were 0.303 and −0.176 Å^−3^.

Crystal data for **7**: C_24_H_29_N_3_O_6_, *M*_r_ = 455.5 g/mol, Monoclinic, Space group *P*2_1_, *a* = 10.844 (1) Å, *b* = 5.702 (6) Å, *c* = 21.029 (2) Å, β = 91.469(2) *Z* = 2, V = 1299.2(4) Å^3^, *D_calc_* = 1.164 Mg/m^3^, F(000) = 484, *R* = 0.056 (reflections collected/unique = 4577/3576), *wR2* = 0.1607, *T* = 294(2)K, and *GOF* = 1.052. The reflections were collected in the range 0.969° ≤ θ ≤ 25.04° (limiting indices = −12 ≤ h ≤ 12, −6 ≤ k ≤ 6, −25 ≤ l ≤ 25) employing a 0.30 × 0.08 × 0.03 mm crystal. The residual positive and negative electron densities in the final map were 0.486 and −0.296 Å^−3^.

### 3.4. Optical Rotatory Power

The values of optical rotations were registered on a polarimeter (mod. 241, PerkinElmer, Waltham, MA, USA) in a 1 dm path length cell at 20 °C, setting the wavelength at 589 nm.

## 4. Conclusions

Although ergometrine and methylergometrine have been known for over 70 years and they have been used as antihemorrhagic agents for a long time, few and sometimes contradictory NMR and crystallographic data are available in the literature. In this work, we provide the complete assignment of ^1^H, ^13^C, and ^15^N NMR signals of ergometrine, methylergometrine, and their maleate salts together with a solid-state analysis of ergometrine maleate and methylergometrine maleate. The new and more accurate X-ray and NMR data will contribute to widen the available information about conformational equilibria and hydrogen-bonding interactions, which could prove useful for the study of other ergot alkaloids.

## Figures and Tables

**Table 1:** ^1^H NMR chemical shifts (ppm) ^a^ and coupling constants (Hz) ^b^ of compounds **4**–**7**.

^1^H	4 (DMSO-*d*_6_)	5 (DMSO-*d*_6_)	6 (D_2_O)	7 (D_2_O)
H-1	10.69 (brs)	10.70 (brs)	−	−
H-2	7.02 (brs)	7.02 (brs, partially overlapped)	7.15 (brs)	7.16 (brs)
H-4α	2.44 (overlapped)	2.45 (overlapped)	2.89 (brt, *J* = 13.1)	2.91 (brt, *J* = 13.1)
H-4β	3.46 (dd, *J* = 14.9, 5.7)	3.46 (dd, *J* = 14.7, 5.5)	3.69 (overlapped)	3.73 (overlapped)
H-5β	3.00–2.93 (m)	3.00–2.93 (m)	3.92 (br)	3.99 (br)
H-7β	2.50 (overlapped with DMSO)	2.50 (overlapped with DMSO)	3.47 (brt, 10.8)	3.49 (brt, *J* = 11.7)
H-7α	3.00 (dd, *J* = 11.0, 5.0)	3.02 (dd, *J* = 11.2, 5.3)	3.77 (br)	3.79 (br)
H-8α	3.40–3.36 (m, overlapped)	3.47–3.42 (m, overlapped)	3.83 (br)	3.89 (overlapped)
H-9	6.34 (brs)	6.35 (brs)	6.46 (brs)	6.49 (brs)
H-12	7.04 (overlapped)	7.03 (brdd, *J* = 8.2, 0.8, partially overlapped)	7.22 (d, *J* = 7.3)	7.23 (d, *J* = 7.1)
H-13	7.06 (tripletoid m)	7.06 (tripletoid m)	7.26 (tripletoid m)	7.27 (tripletoid m)
H-14	7.18 (dd, *J* = 6.9, 1.1)	7.18 (dd, *J* = 7.4, 0.9)	7.42 (d, *J* = 7.8)	7.43 (d, *J* = 7.6)
N-CH_3_	2.44 (s)	2.45 (s)	3.11 (s)	3.13 (s)
CH	3.86–3.76 (m)	3.71–3.62 (m)	4.10-4.02 (m)	3.94–3.84 (m)
CH_3_	1.05 (d, *J* = 6.6)	0.85 (t, *J* = 7.4)	1.21 (d, *J* = 6.9)	0.95 (t, *J* = 7.1)
C*H*_a_CH_3_	−	1.39–1.26 (m)	−	1.54–1.42 (m)
C*H*_b_CH_3_	−	1.66–1.53 (m)	−	1.73–1.62 (m)
C*H*_a_OH	3.32–3.25 (m)	3.37–3.30 (m)	3.61 (dd, *J* = 11.5, 6.6)	3.63 (dd, *J* = 11.7, 6.6)
C*H*_b_OH	3.43–3.36 (m)	3.42–3.38 (m)	3.71 (dd, *J* = 11.7, 6.0)	3.73 (dd, *J* = 11.5, 4.6)
CH_2_O*H*	4.72 (brs)	4.68 (t, *J* = 5.7)	−	−
CON*H*	7.76 (d, *J*=7.8)	7.69 (d, *J* = 8.5)	−	−
CH=CH maleate	−	−	6.21 (s)	6.23 (s)

^a^ Assignments from ^1^H–^1^H COSY, NOESY, and HSQC data. ^b^ Coupling constants were obtained by direct inspection of the spectra. Error in the measured ^1^H-^1^H coupling constants was ± 0.5 Hz.

**Table 2:** ^13^C NMR chemical shifts (ppm) ^a^ of compounds **4**–**7**.

^13^C	4 (DMSO-*d*_6_)	5 (DMSO-*d*_6_)	6 (D_2_O)	7 (D_2_O)
C-2	119.2	119.2	121.5	121.5
C-3	109.0	109.0	106.4	106.4
C-4	26.8	26.8	24.8	24.7
C-5	62.6	62.6	62.8	62.7
C-7	55.5	55.7	54.1	54.1 (overlapped)
C-8	42.8	42.9	41.4	41.5
C-9	120.3	120.4	118.7	118.7
C-10	135.1	135.0	132.6	132.6
C-11	127.4	127.5	125.1	125.2
C-12	111.0	111.0	113.2	113.2
C-13	122.2	122.3	124.0	124.0
C-14	109.7	109.7	112.2	112.2
C-15	133.8	133.9	134.3	134.3
C-16	125.8	125.8	125.7	125.7
N-CH_3_	43.4	43.4	42.1	42.0
CH	46.5	52.1	48.3	54.2 (overlapped)
CH_3_	17.2	10.5	16.5	10.3
*C*H_2_CH_3_	−	23.7	−	24.0
CH_2_OH	64.4	63.0	65.0	63.8
CH=CH maleate	−	−	134.9	134.9
COOH	−	−	171.5	171.6
CONH	171.2	171.7	171.8	172.4

^a^ Assignments from HSQC and HMBC data.

**Table 3:** ^15^N NMR chemical shifts (ppm) ^a^ of compounds **4**–**7**.

	4 (DMSO-*d*_6_)	5 (DMSO-*d*_6_)	6 (D_2_O)	7 (D_2_O)
N-1	−251.1 (128.4)	−251.0 (128.5)	−253.3 (126.2)	−253.3 (126.2)
N-6	−340.3 (39.2)	−340.2 (39.3)	−332.8 (46.7)	−332.7 (46.8)
CONH	−255.5 (124.0)	−258.1 (121.4)	−248.3 (131.2)	−251.4 (128.1)

^a^ Assignments from ^1^H-^15^N HMBC data using nitromethane as the external reference (CH_3_NO_2_ δ = 0.00 ppm). In parenthesis ^15^N chemical shifts referred to ammonia (δ_NH__3_ = −379.5 ppm respect to CH_3_NO_2_).

**Table 4:** Long range couplings of hydrogen with quaternary carbon atoms observed in the HMBC spectra of compounds **4**–**7**.

Quaternary Carbon	HMBC (C→H)	
	4 and 5 (DMSO-*d*_6_)	6 and 7 (D_2_O)
C-3	H-4α, H-4β, H-2, H-1	H-4α, H-2
C-10	H-13, H-12, H-5, H-4α, H-4β	H-13, H-12, H-4α
C-11	H-13, H-12, H-9	H-13, H-9
C-15	H-14, H-13, H-2, H-1	H-13, H-12, H-2
C-16	H-14, H-12, H-4β, H-2, H-1	H-14, H-12, H-4α, H-2

**Table 5:** Hydrogen-bonds geometry of **6** and **7** (arbitrary atom-numbering scheme used in Figure 4).

	6			7		
H-Bond	H···A Distance (Å)	D···A Distance (Å)	D-H···A Angle (°)	H···A Distance (Å)	D···A Distance (Å)	D-H···A Angle (°)
N2-H···O3	1.8(1)	2.7(1)	176(1)	1.8(1)	3.2(1)	168(1)
N1-H···O2 ^I^	2.9(1)	3.5(1)	140(1)	-		-
C22-H···O6 ^II^	2.7(1)	3.3(1)	134(1)	2.7(1)^V^	3.3(1)	130(1)
C21-H···O4 ^II^	2.8 (1)	3.7(1)	171(1)	-		-
C21-H···O6 ^II^	2.7(1)	3.4(1)	132(1)	2.6 (1) ^V^	3.3(1)	133(1)
O2-H···O1 ^II^	2.2(1)	2.8(1)	131(1)	-		-
N1-H···O1 ^III^	2.1(1)	2.8(1)	155(1)	-		-
N3-H···O5 ^IV^	2.1(1)	2.9(1)	163(1)	2.1(1) ^VI^	2.(1)	172(1)

Equivalent positions: ^I^ −x, y − ½, ½ − z; ^II^ x − 1, y, z; ^III^ 1 − x, y − ½, ½ − z; ^IV^ x − ½, ½ − y, −z; ^V^ x, y + 1, z; ^VI^ −x, y + ½, 1 − z.

## Supplementary Materials

The following are available online, The NMR spectra (^1^H, ^13^C, COSY, HSQC, ^1^H-^13^C HMBC, ^1^H-^15^N HMBC and NOESY) of compounds **4**–**7** are available online.
