# Supplementary material for: Crystallographic and NMR Investigation of Ergometrine and Methylergometrine, Two Alkaloids from Claviceps Purpurea
Source: Molecules. 2020 Jan 14;25(2):331. doi: 10.3390/molecules25020331 (PMC7024318; doi:10.3390/molecules25020331)
Supplement: Supplementary file 1 [file molecules-25-00331-s001.pdf]

Crystallographic and NMR Investigation on Ergometrine and Methylergometrine, Two Alkaloids from *Claviceps purpurea*

Fiorella Meneghetti [1], Patrizia Ferraboschi [2], Paride Grisenti [3], Shahrzad Reza Elahi [2], Matteo Mori [1] and Samuele Ciceri [2,*]

[1] Department of Pharmaceutical Sciences "Pietro Pratesi", University of Milan, via L. Mangiagalli, 25 - 20133 Milano Italy; fiorella.meneghetti@unimi.it (F.M.); matteo.mori@unimi.it (M.M.)

[2] Department of Medical Biotechnology and Translational Medicine, University of Milan, Via Saldini 50, 20133 Milano, Italy; shahrzad.rezaelahi@gmail.com (S.R.); patrizia.ferraboschi@unimi.it (P.F.)

[3] Chemical-Pharmaceutical Consulting and IP Management, Viale Giovanni da Cermenate 58, 20141 Milano, Italy; grisenti.paride60@gmail.com (P.G.)

* Correspondence: samuele.ciceri@guest.unimi.it (S.C.)

Supplementary data contents

S3 ^{1}H NMR of ergometrine maleate **6**
S4 ^{13}C NMR of ergometrine maleate **6**
S5 COSY of ergometrine maleate **6**
S6 HSQC of ergometrine maleate **6**
S7 ^{1}H-^{13}C HMBC of ergometrine maleate **6**
S8 NOESY of ergometrine maleate **6**
S9 ^{1}H-^{15}N HMBC of ergometrine maleate **6**
S10 ^{1}H NMR of methylergometrine maleate **7**
S11 ^{13}C NMR of methylergometrine maleate **7**
S12 COSY of methylergometrine maleate **7**
S13 HSQC of methylergometrine maleate **7**
S14 ^{1}H-^{13}C HMBC of methylergometrine maleate **7**
S15 NOESY of methylergometrine maleate **7**
S16 ^{1}H-^{15}N HMBC of methylergometrine maleate **7**
S17 ^{1}H NMR of ergometrine **4**
S18 ^{13}C NMR of ergometrine **4**
S19 COSY of ergometrine **4**
S20 HSQC of ergometrine **4**
S21 ^{1}H-^{13}C HMBC of ergometrine **4**
S22 NOESY of ergometrine **4**
S23 NOESY of ergometrine **4** (expansion of the 2.3 ÷ 3.6 ppm region)
S24 ^{1}H-^{15}N HMBC of ergometrine **4**
S25 ^{1}H NMR of methylergometrine **5**
S26 ^{13}C NMR of methylergometrine **5**
S27 COSY of methylergometrine **5**
S28 HSQC of methylergometrine **5**
S29 ^{1}H-^{13}C HMBC of methylergometrine **5**
S30 NOESY of methylergometrine **5**
S31 NOESY of methylergometrine **5** (expansion of the 2.3 ÷ 3.6 ppm region)
S32 ^{1}H-^{15}N HMBC of methylergometrine **5**

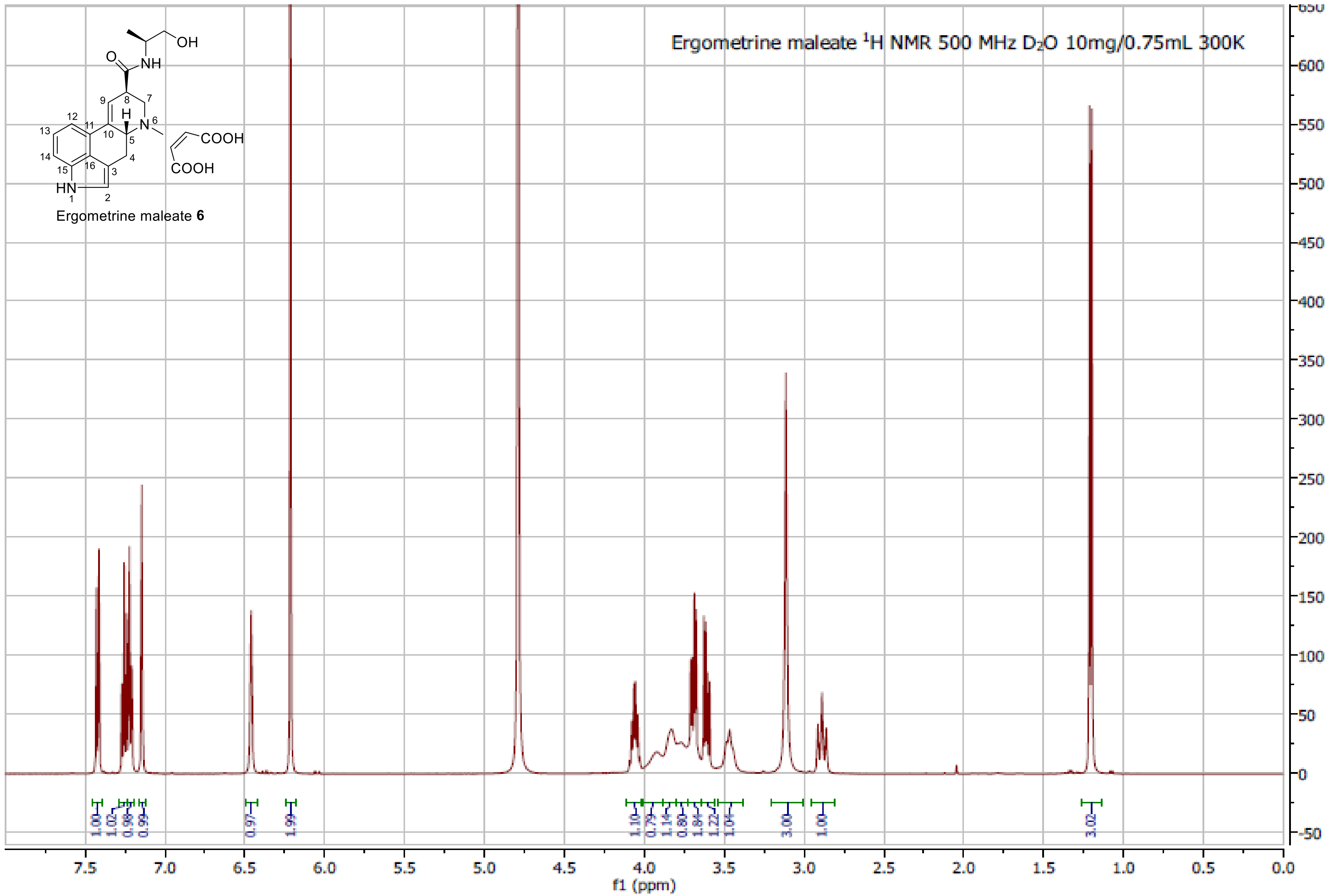

Ergometrine maleate 6
Ergometrine maleate ¹H NMR 500 MHz D₂O 10mg/0.75mL 300K
f1 (ppm)

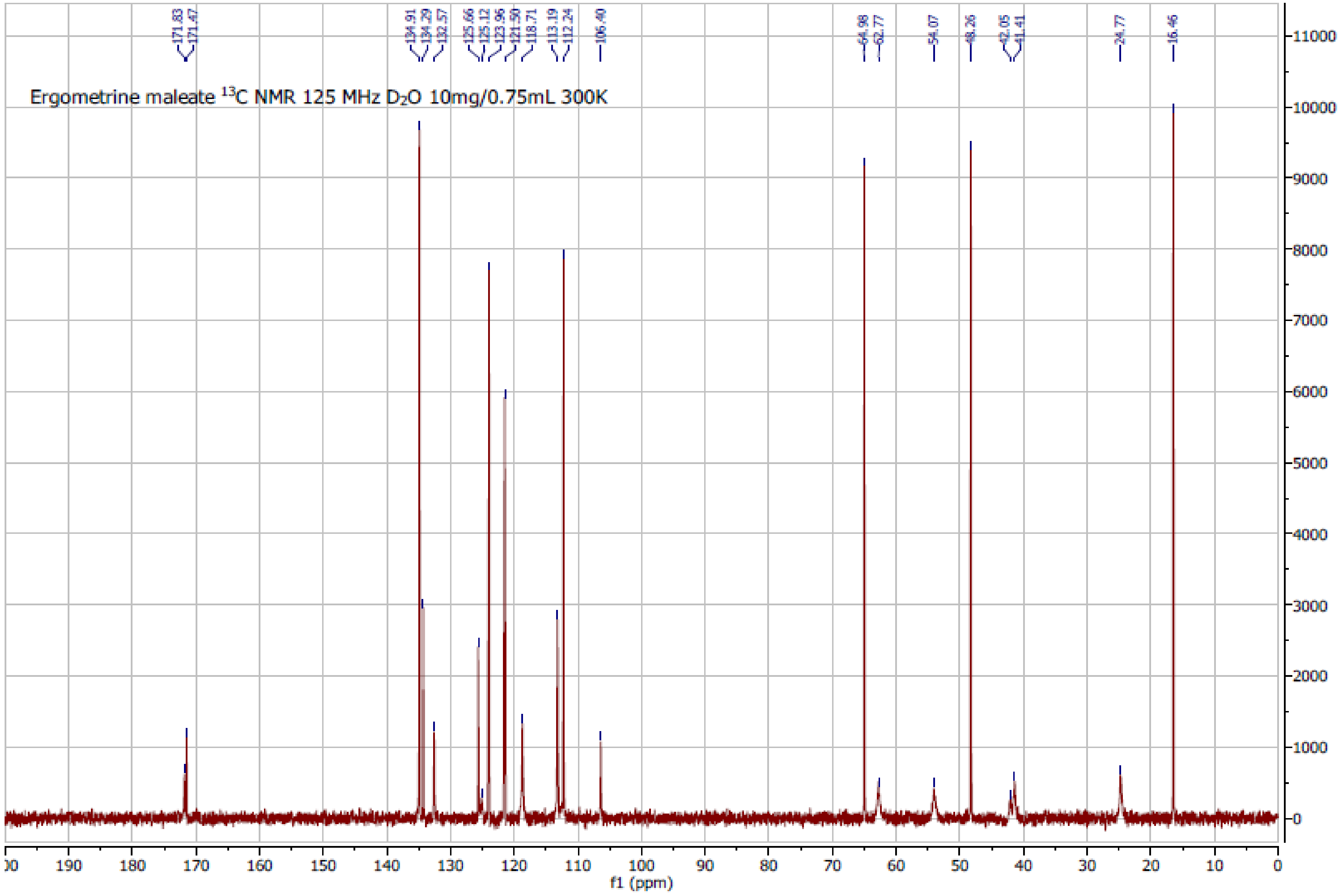
Ergometrine maleate ^{13}C NMR 125 MHz D$_2$O 10mg/0.75mL 300K
171.83
171.47
134.91
134.29
132.57
125.66
125.12
123.96
121.50
118.71
113.19
112.24
106.40
64.98
62.77
54.07
48.26
42.05
41.41
24.77
16.46
f1 (ppm)

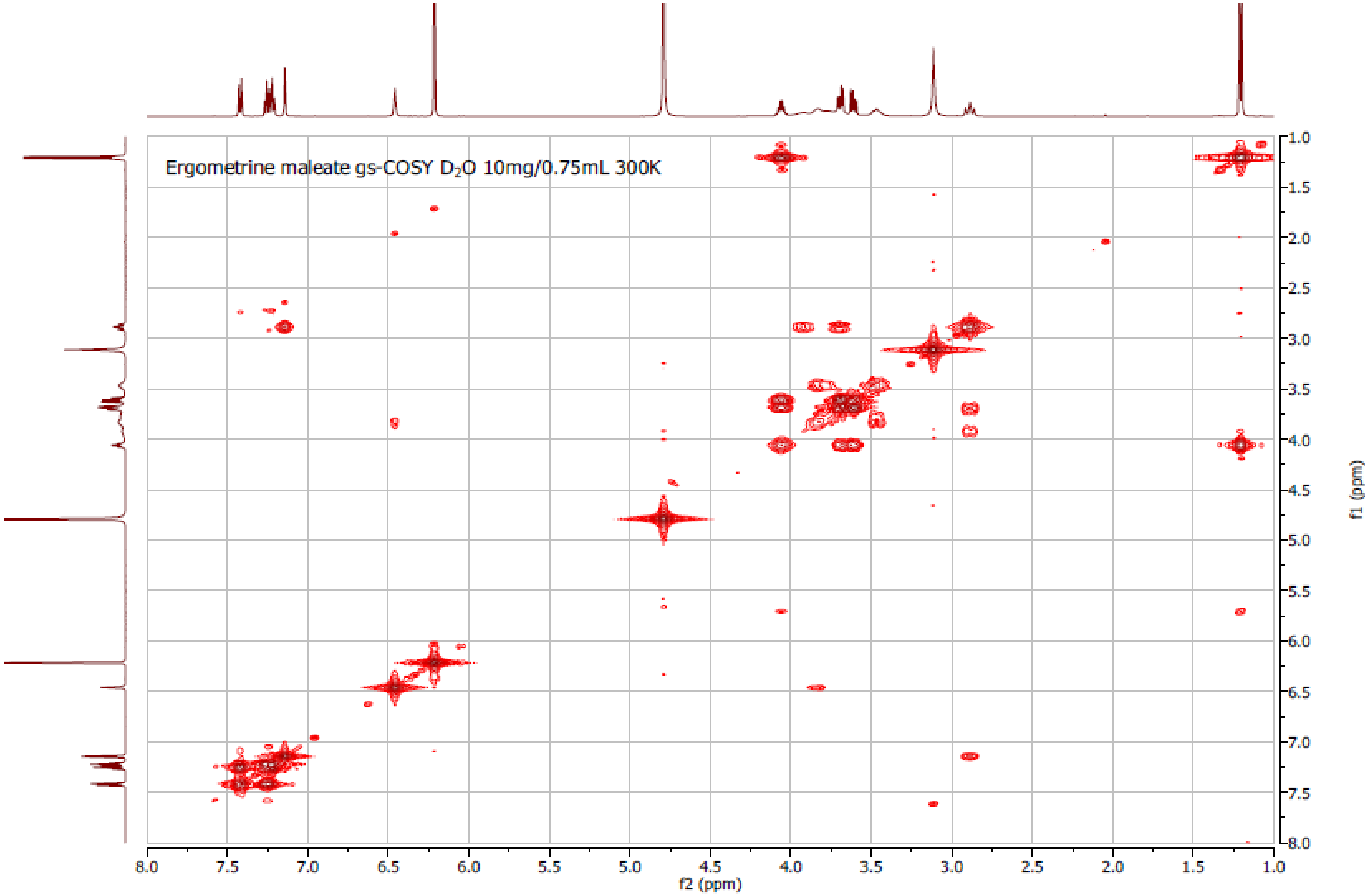

Ergometrine maleate gs-COSY D_2O 10mg/0.75mL 300K
f2 (ppm)
f1 (ppm)

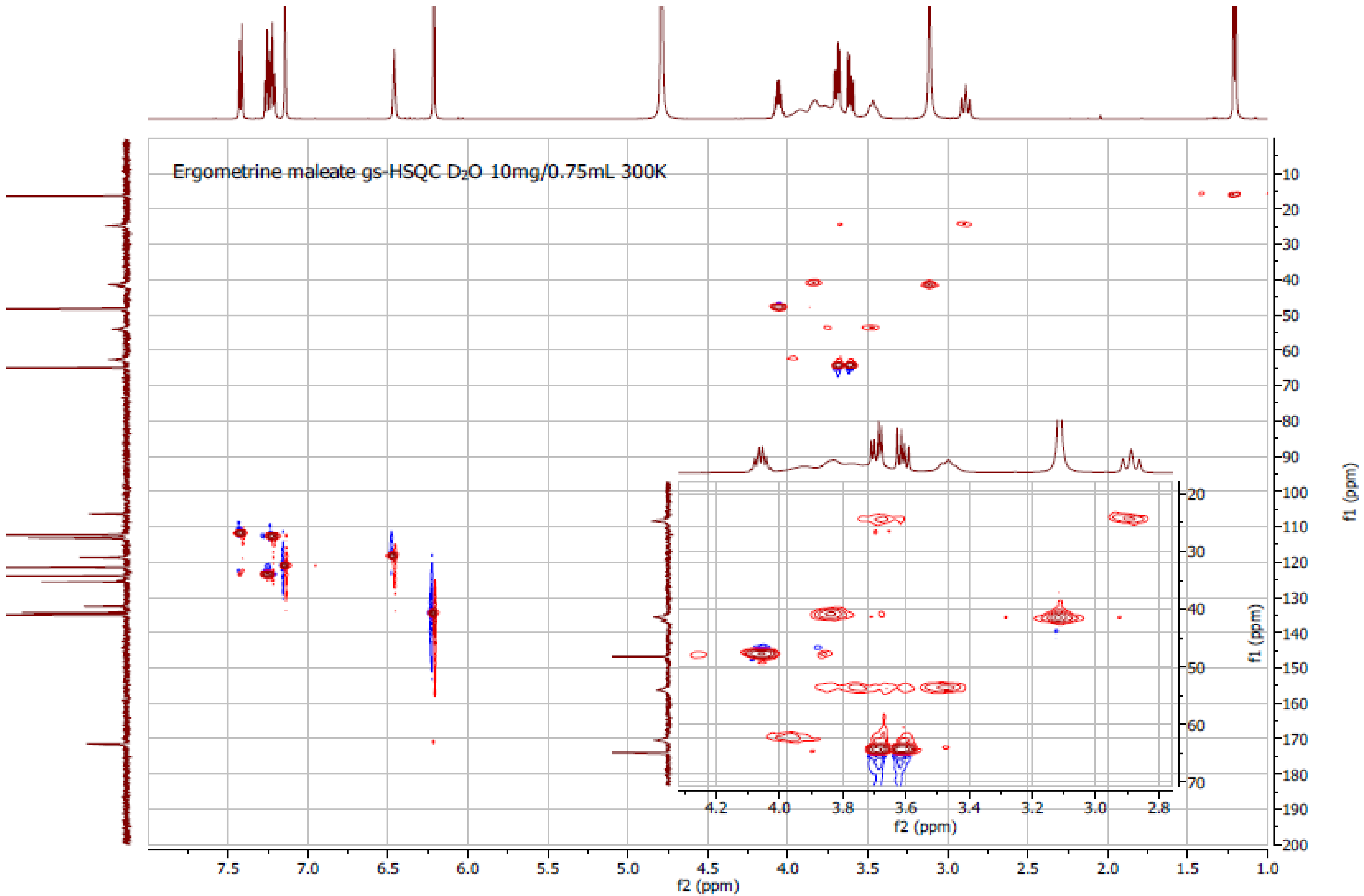

Ergometrine maleate gs-HSQC D$_2$O 10mg/0.75mL 300K
f2 (ppm)
f1 (ppm)

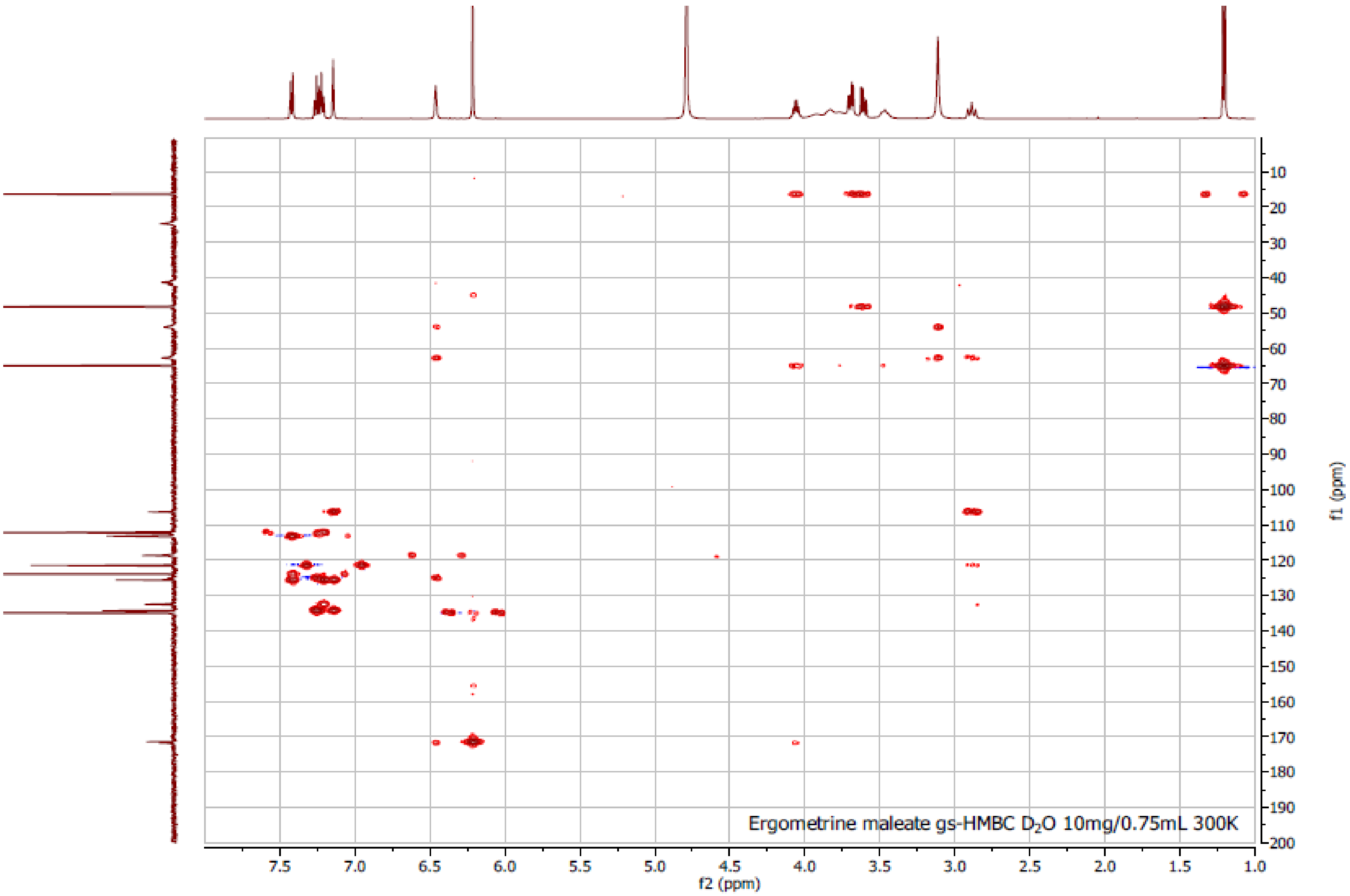

Ergometrine maleate gs-HMBC D_2O 10mg/0.75mL 300K
f2 (ppm)
f1 (ppm)
7.5
7.0
6.5
6.0
5.5
5.0
4.5
4.0
3.5
3.0
2.5
2.0
1.5
1.0
10
20
30
40
50
60
70
80
90
100
110
120
130
140
150
160
170
180
190
200

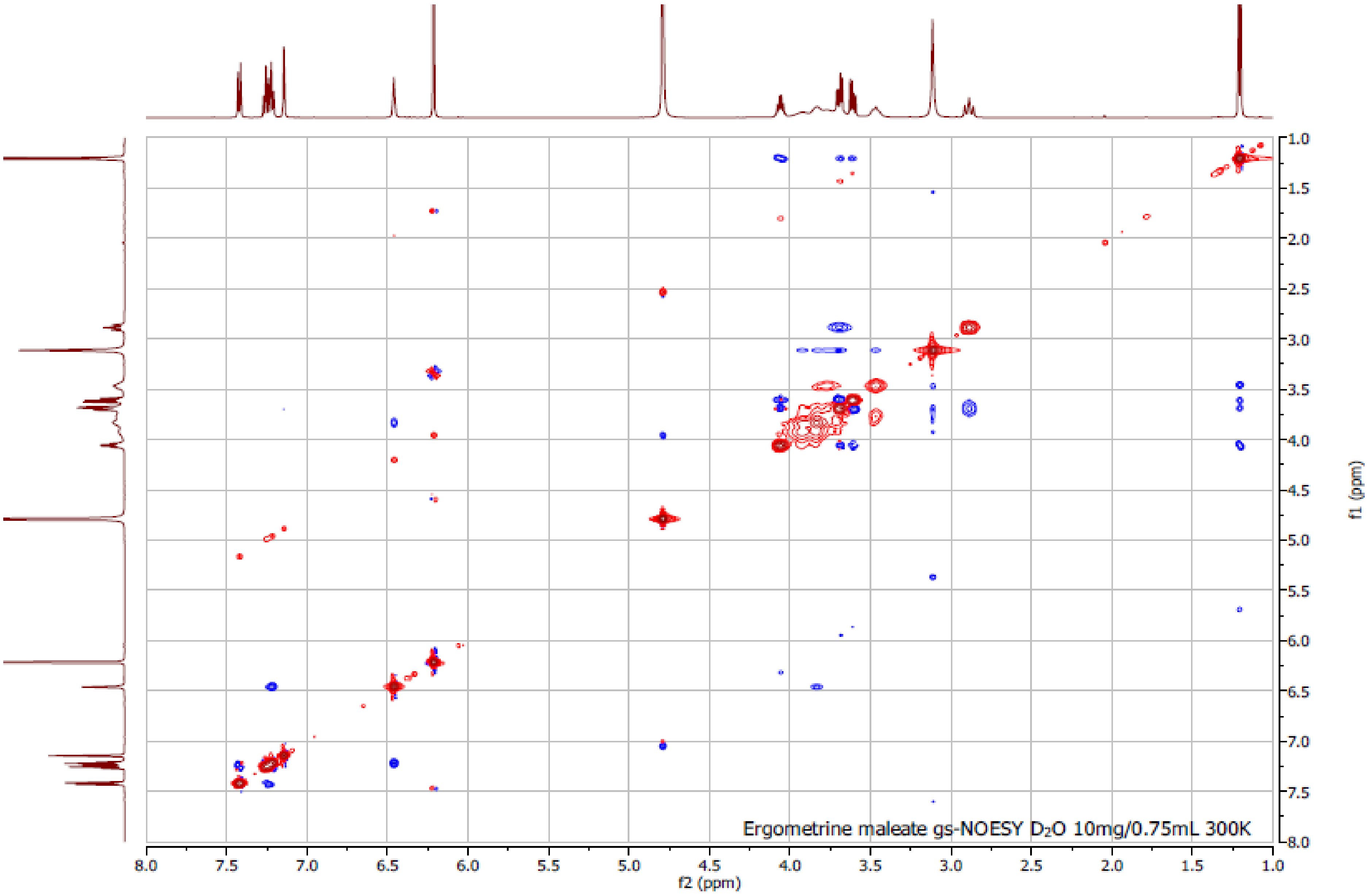

Ergometrine maleate gs-NOESY D₂O 10mg/0.75mL 300K
f2 (ppm)
f1 (ppm)
8.0
7.5
7.0
6.5
6.0
5.5
5.0
4.5
4.0
3.5
3.0
2.5
2.0
1.5
1.0

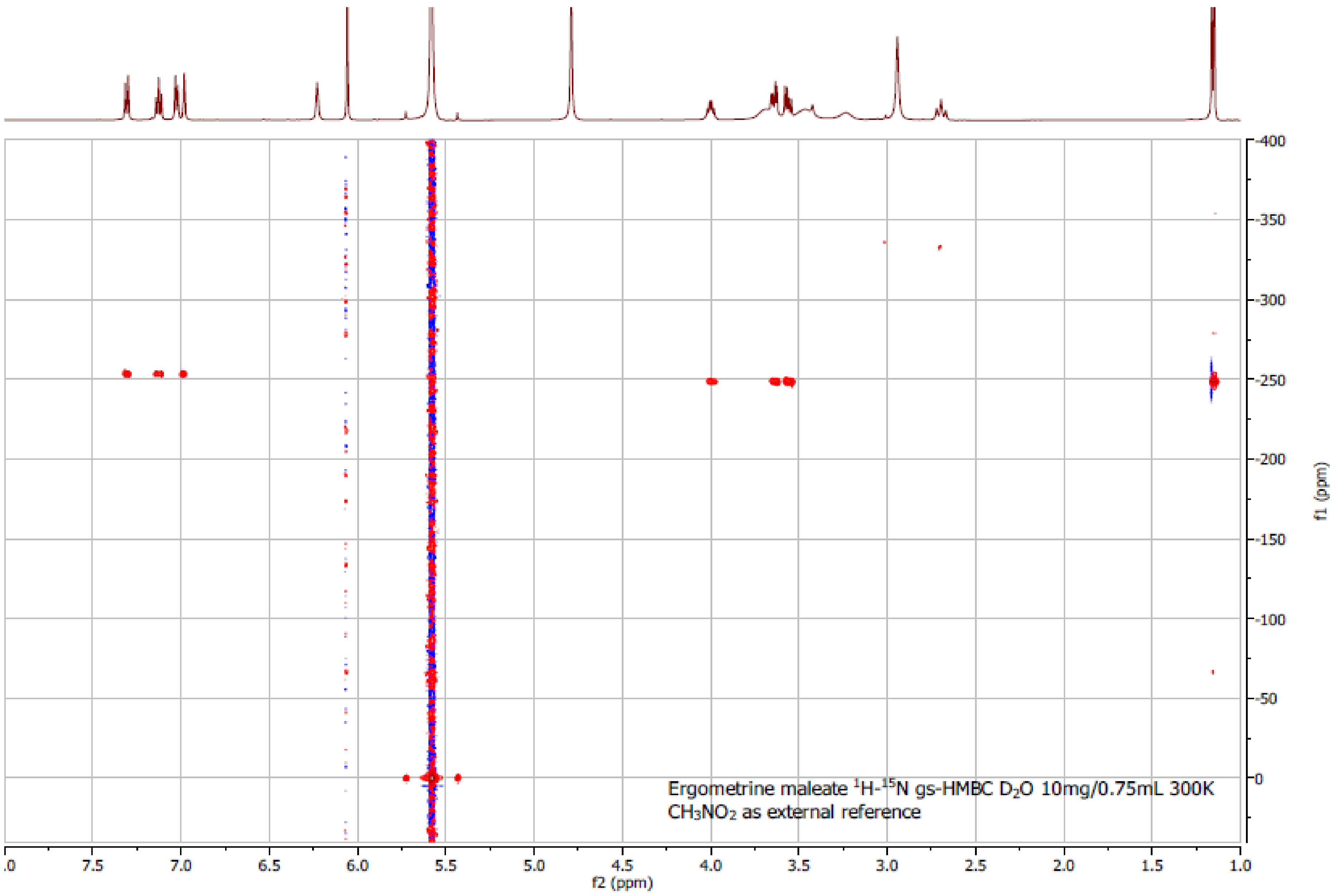
Ergometrine maleate ^{1}H-^{15}N gs-HMBC D_2O 10mg/0.75mL 300K
CH_3NO_2 as external reference
f2 (ppm)
f1 (ppm)

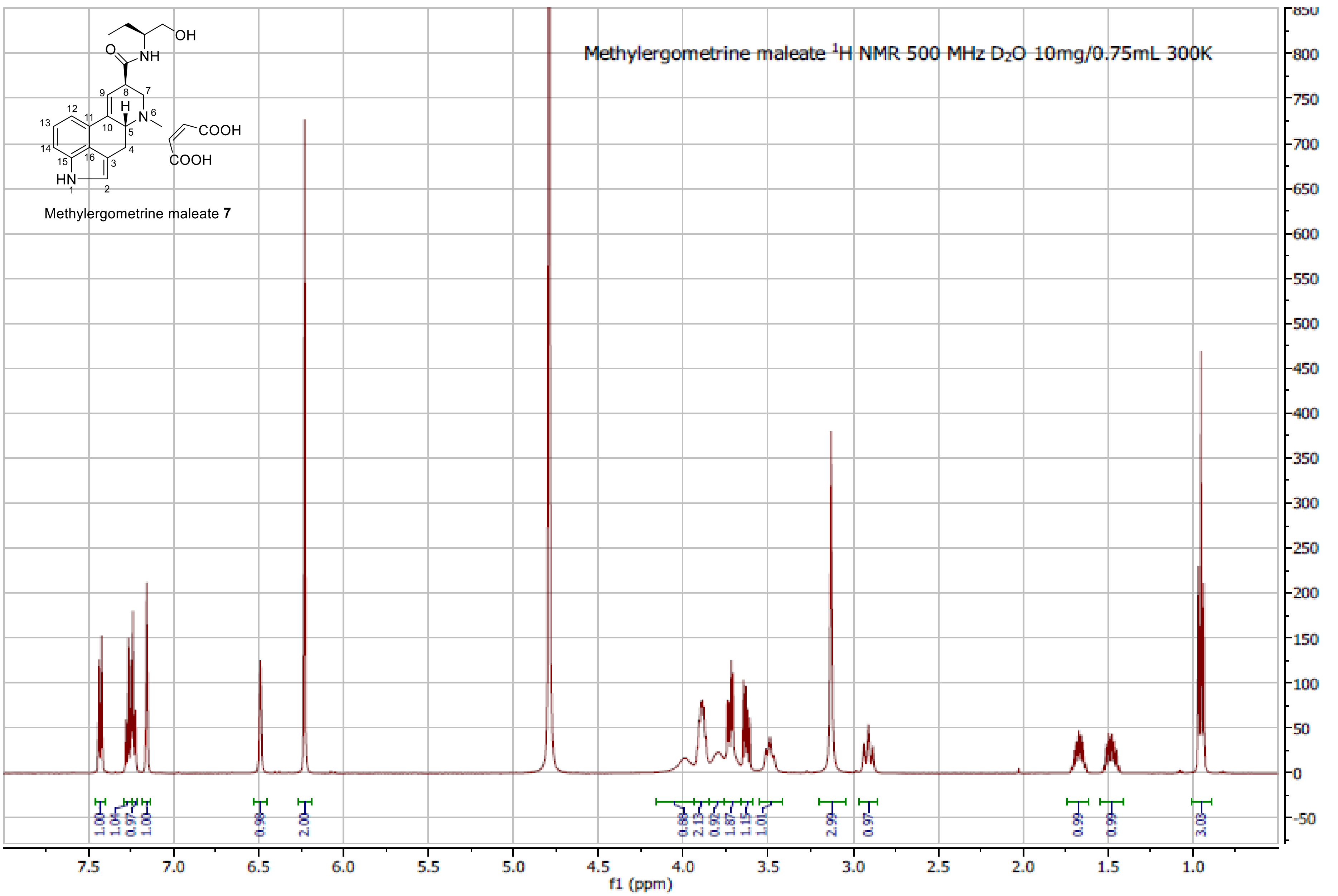
Methylergometrine maleate ^{1}H NMR 500 MHz D_2O 10mg/0.75mL 300K
Methylergometrine maleate **7**
OH
NH
O
HN
COOH
COOH
f1 (ppm)
7.5
7.0
6.5
6.0
5.5
5.0
4.5
4.0
3.5
3.0
2.5
2.0
1.5
1.0
1.00
1.04
0.97
1.00
0.98
2.00
0.80
2.13
0.92
1.87
1.15
1.01
2.99
0.97
0.99
0.99
3.03

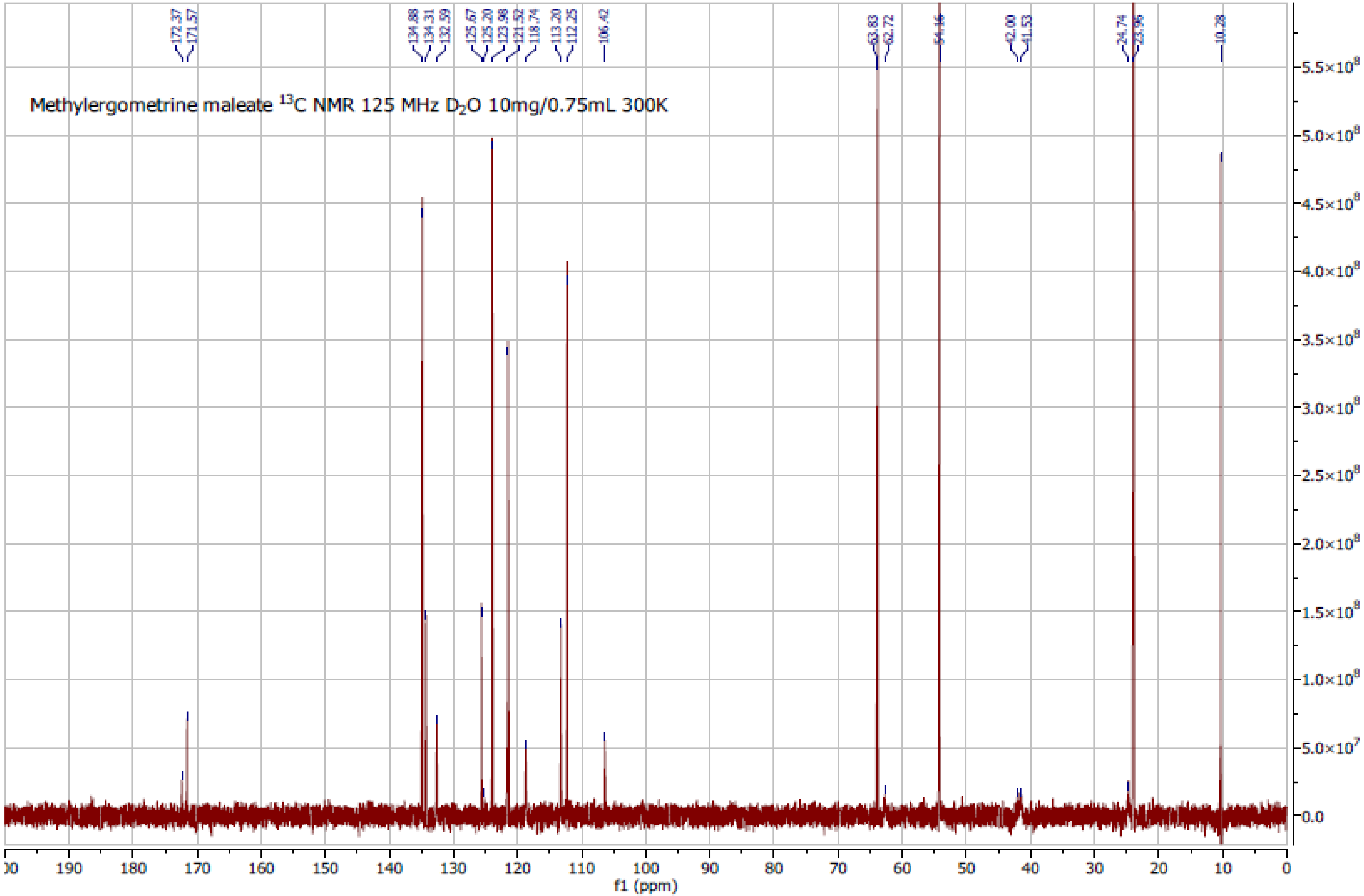

Methylergometrine maleate ^{13}C NMR 125 MHz D_2O 10mg/0.75mL 300K
172.37
171.57
134.88
134.31
132.59
125.67
125.20
123.98
121.52
118.74
113.20
112.25
106.42
63.83
62.72
54.16
42.00
41.53
24.74
23.96
10.28
f1 (ppm)

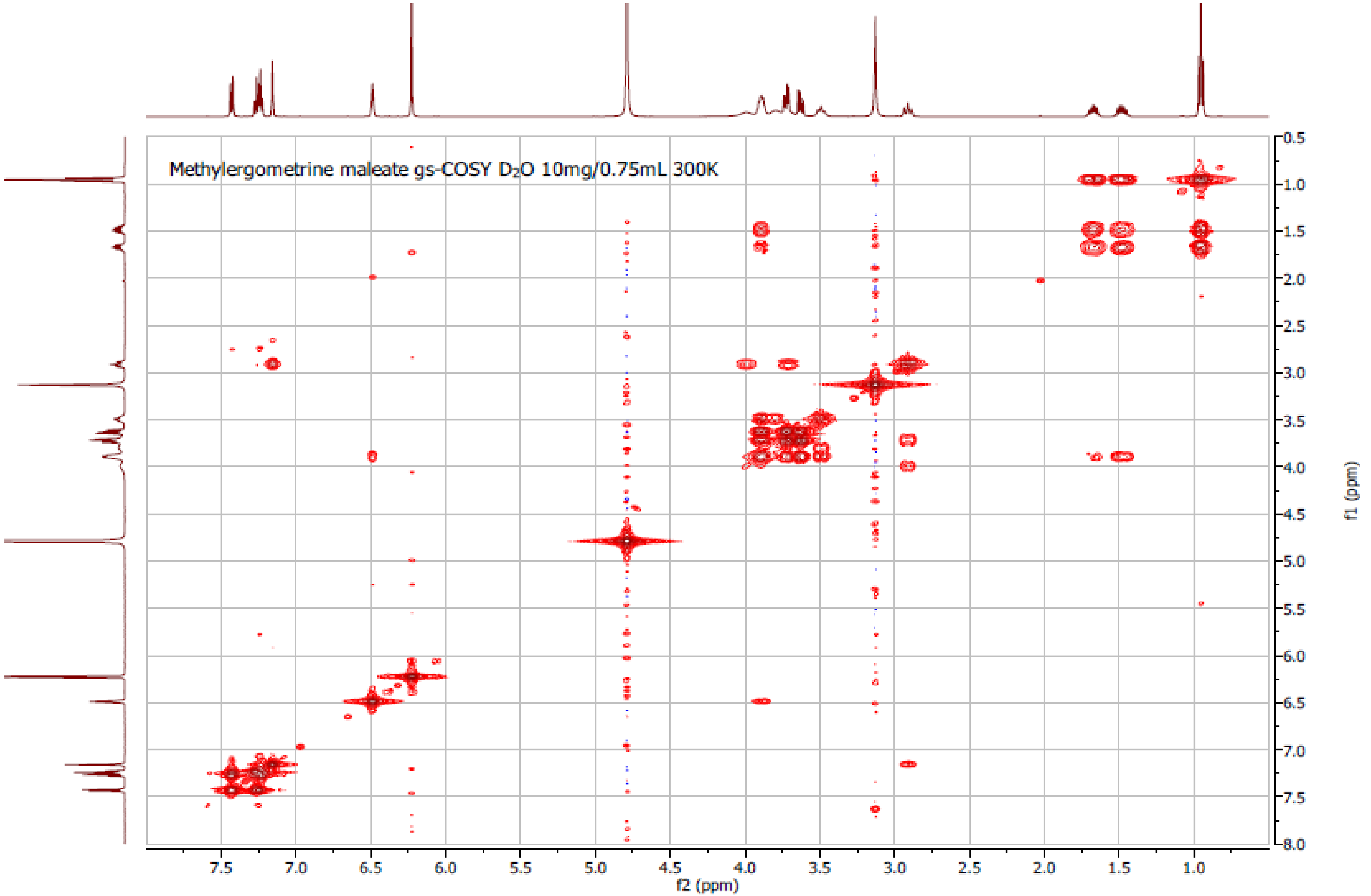
Methylergometrine maleate gs-COSY D₂O 10mg/0.75mL 300K
f2 (ppm)
f1 (ppm)

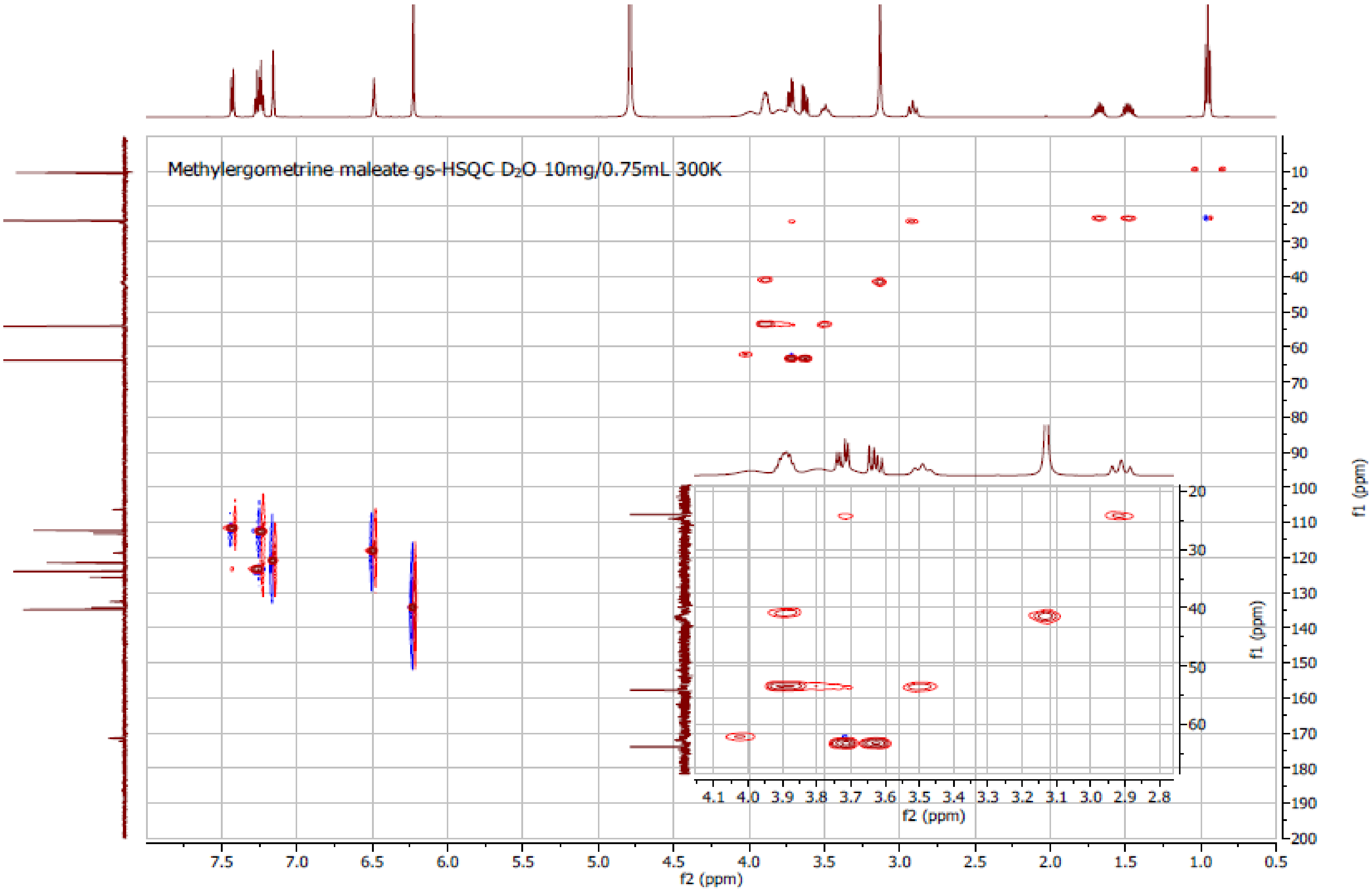
Methylergometrine maleate gs-HSQC D₂O 10mg/0.75mL 300K
f2 (ppm)
f1 (ppm)
f2 (ppm)
f1 (ppm)

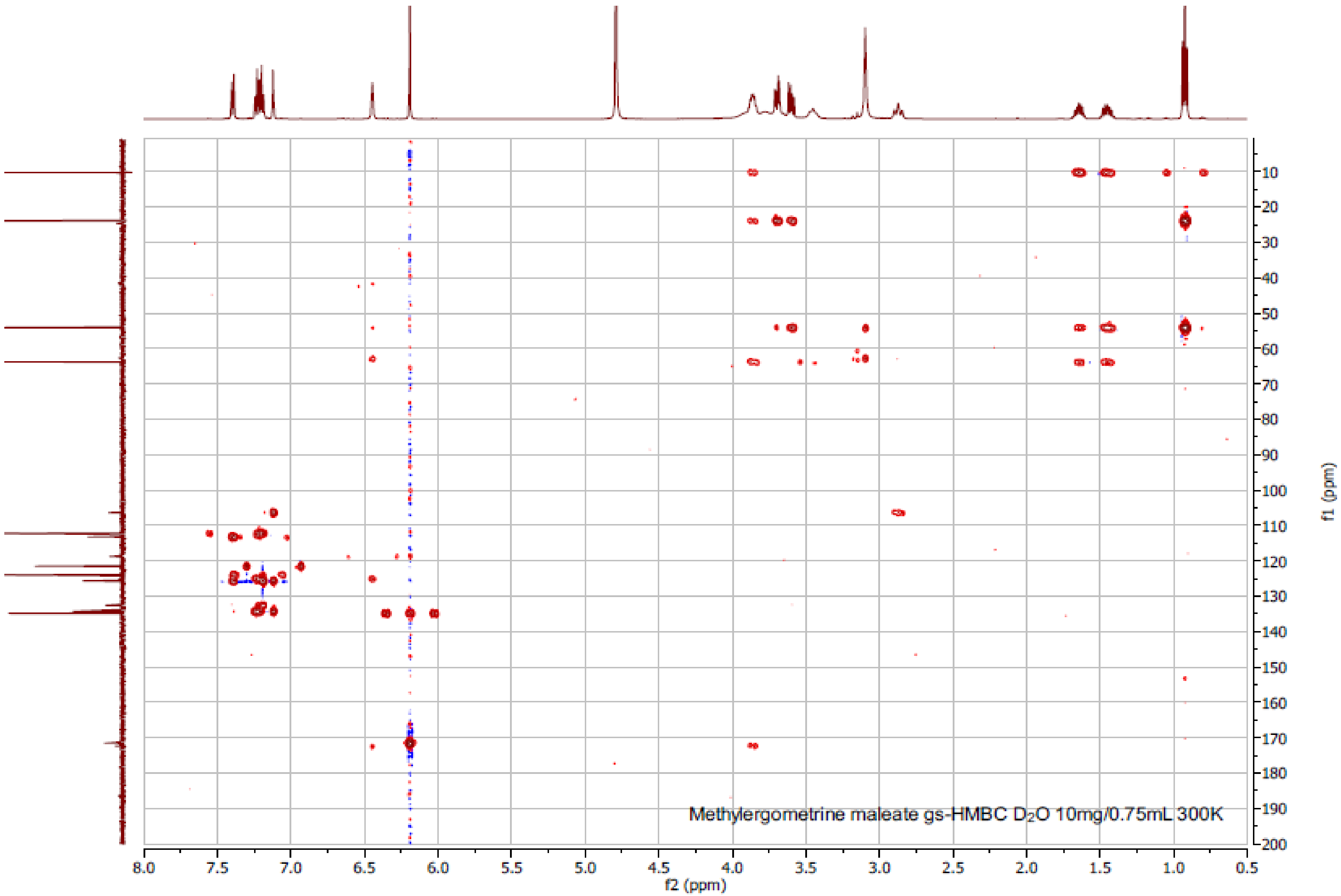

Methylergometrine maleate gs-HMBC D_2O 10mg/0.75mL 300K
f2 (ppm)
f1 (ppm)
8.0
7.5
7.0
6.5
6.0
5.5
5.0
4.5
4.0
3.5
3.0
2.5
2.0
1.5
1.0
0.5
10
20
30
40
50
60
70
80
90
100
110
120
130
140
150
160
170
180
190
200

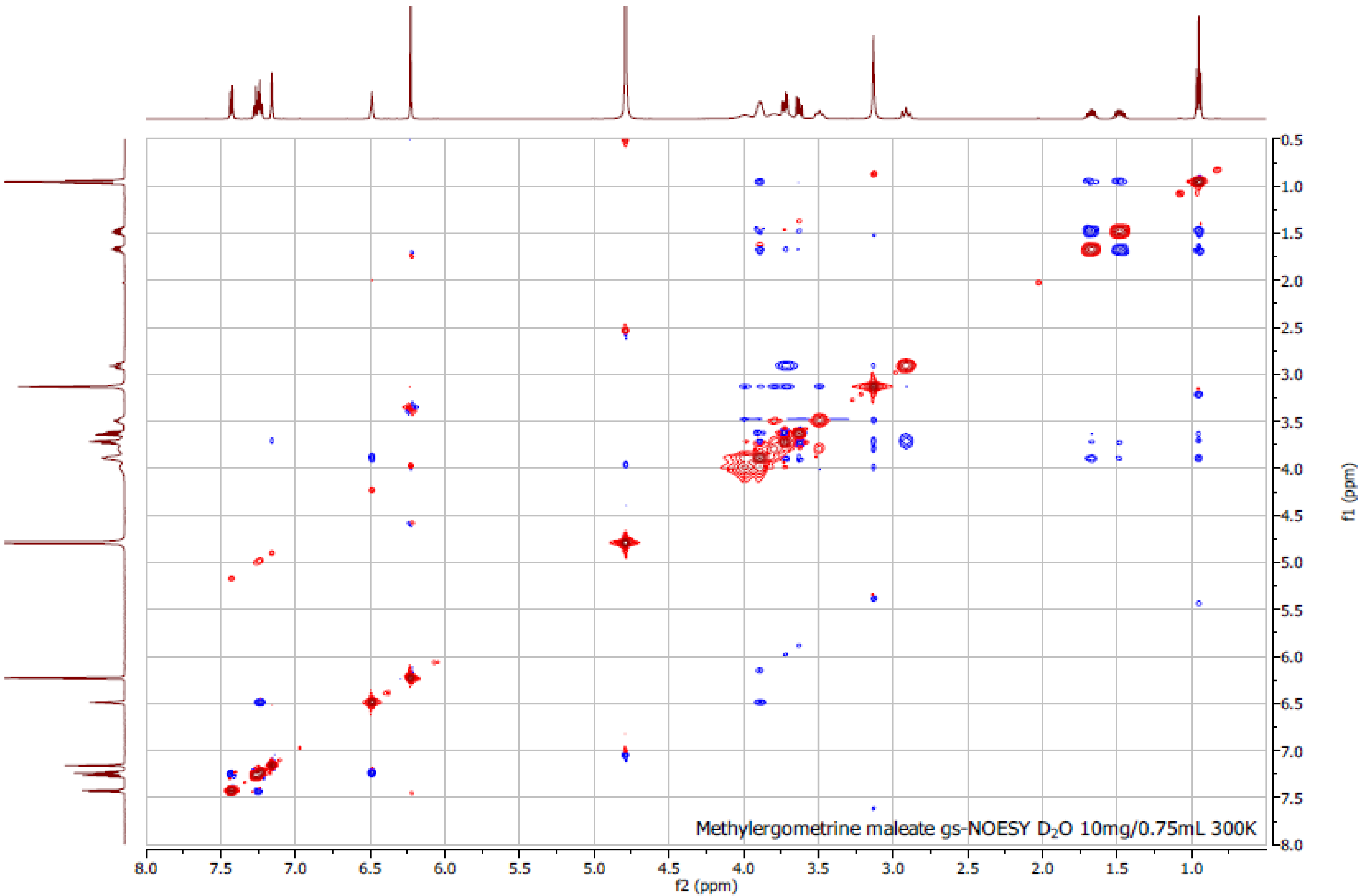
Methylergometrine maleate gs-NOESY D2O 10mg/0.75mL 300K
f2 (ppm)
f1 (ppm)

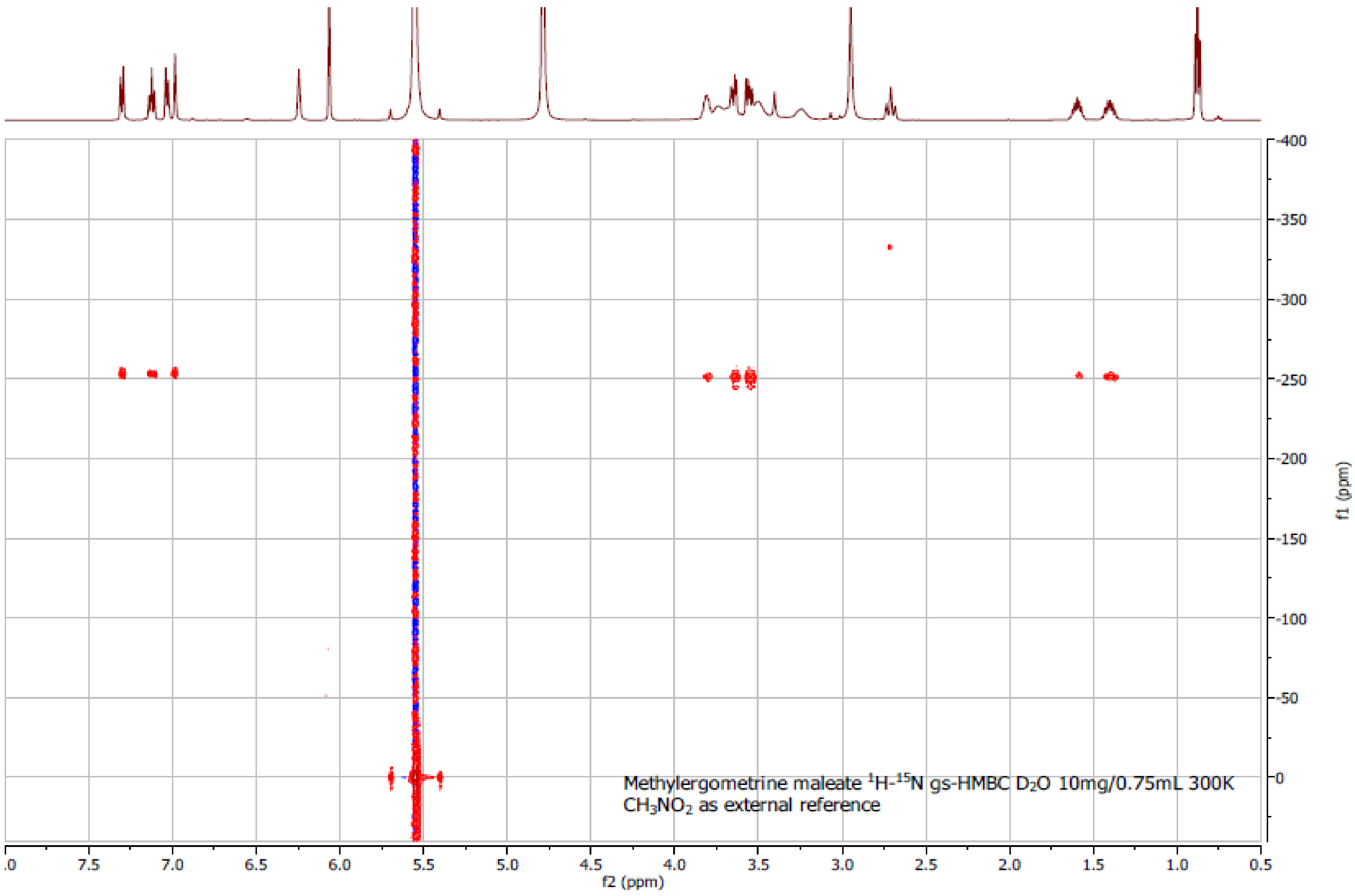
Methylergometrine maleate ^{1}H-^{15}N gs-HMBC D_2O 10mg/0.75mL 300K
CH_3NO_2 as external reference
f2 (ppm)
f1 (ppm)

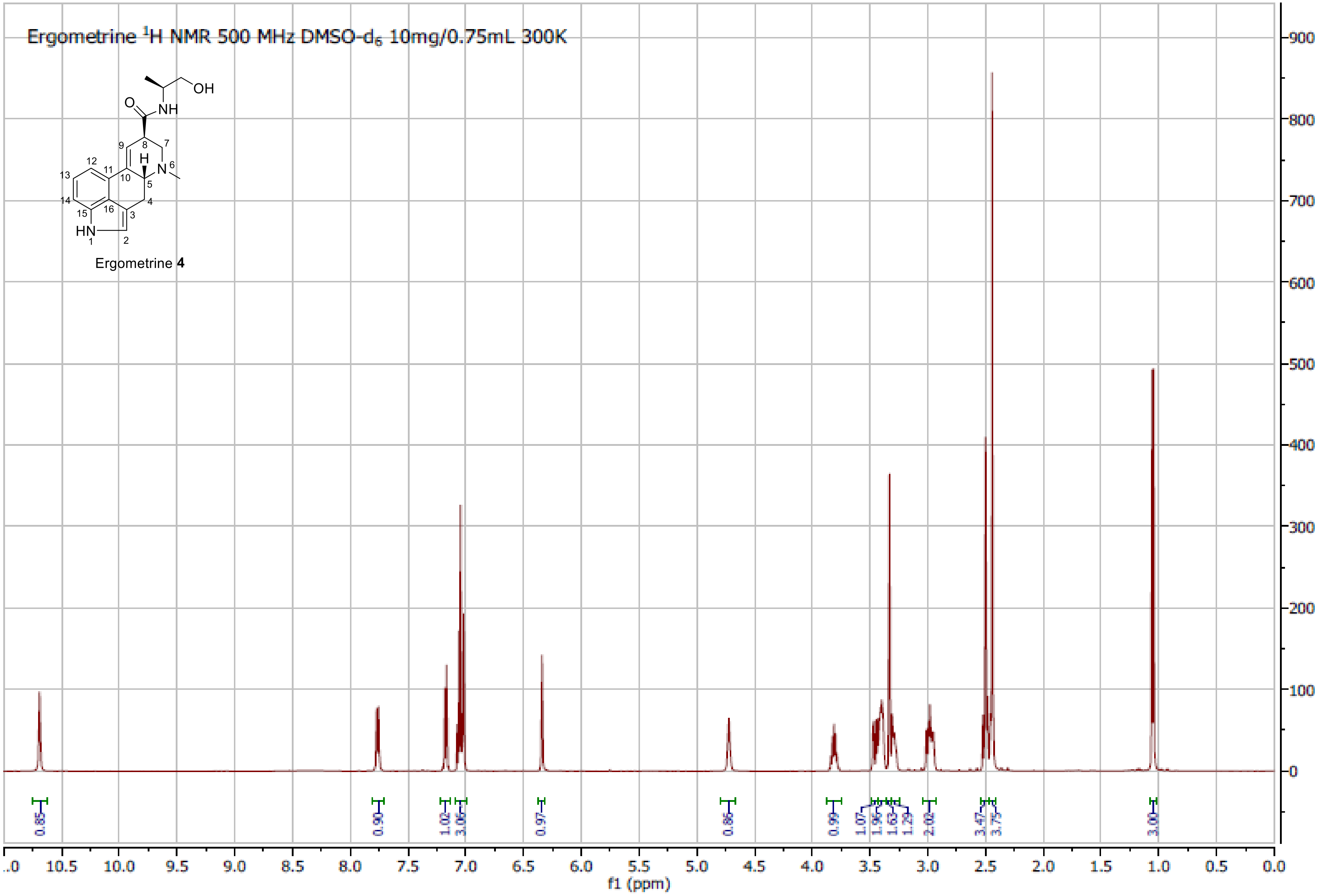
Ergometrine ¹H NMR 500 MHz DMSO-d6 10mg/0.75mL 300K
Ergometrine 4
f1 (ppm)

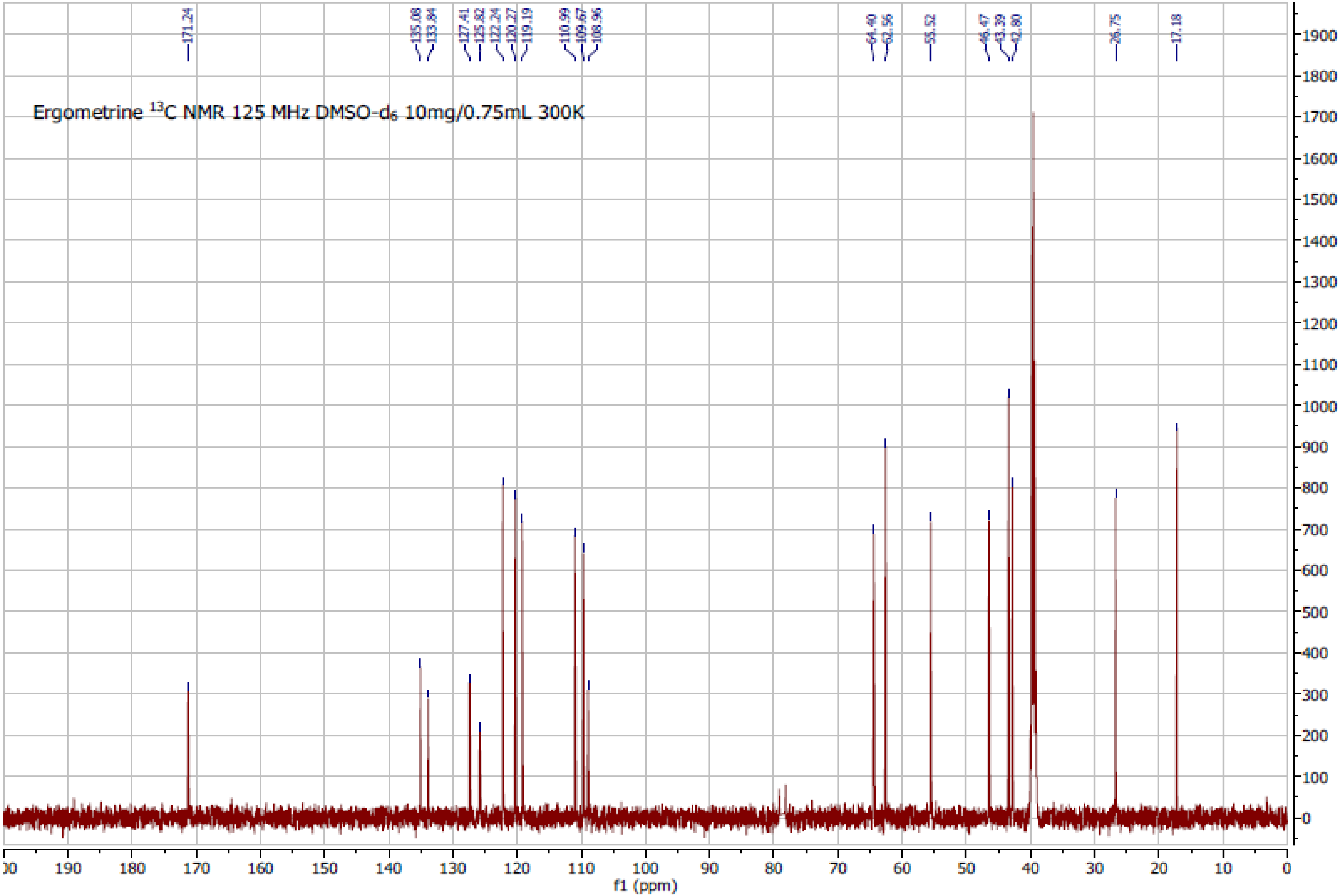

171.24
135.08
133.84
127.41
125.82
122.24
120.27
119.19
110.99
109.67
108.96
64.40
62.56
55.52
46.47
43.39
42.80
26.75
17.18
Ergometrine ¹³C NMR 125 MHz DMSO-d6 10mg/0.75mL 300K
f1 (ppm)

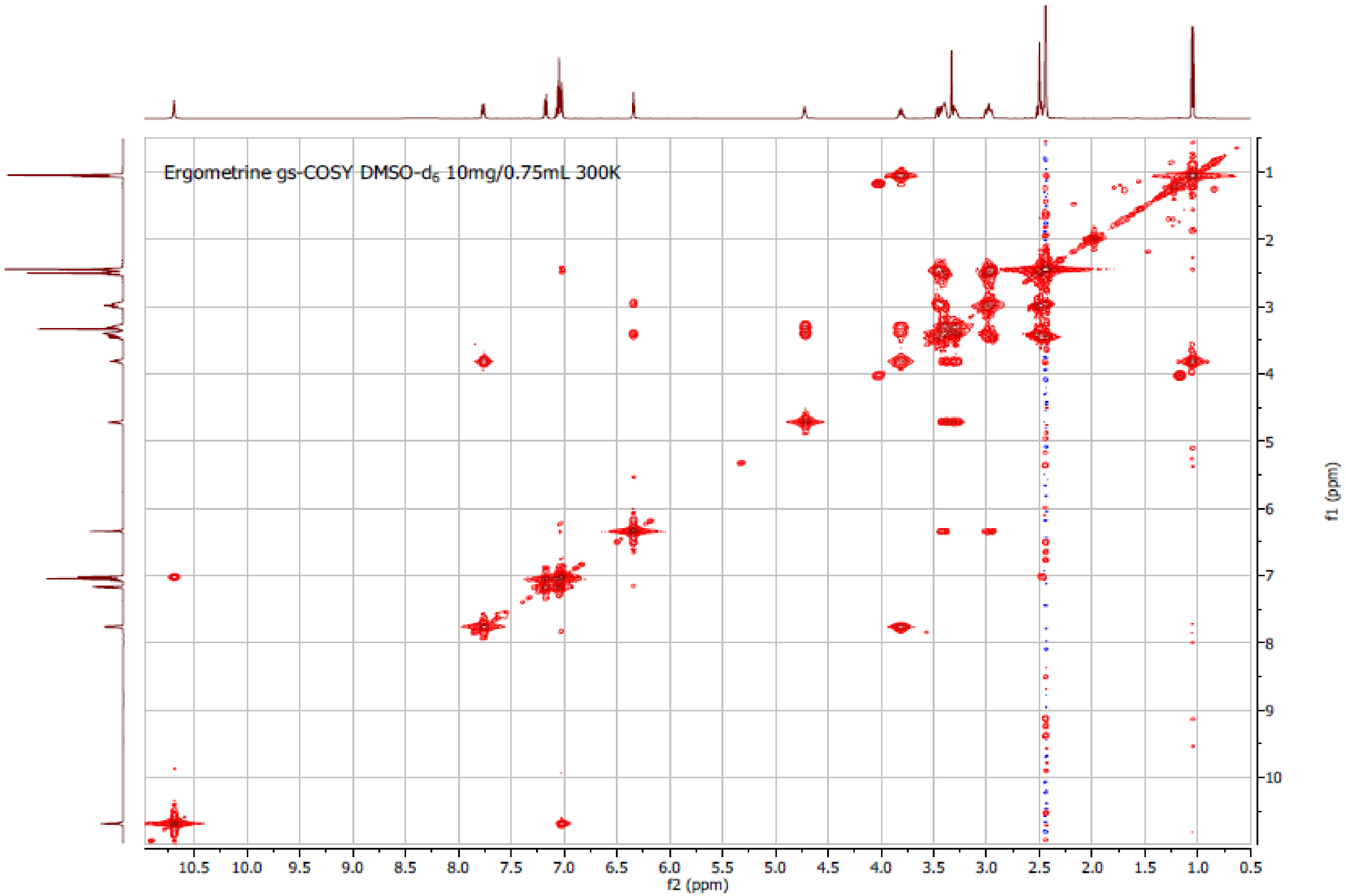

Ergometrine gs-COSY DMSO-d_6 10mg/0.75mL 300K
f2 (ppm)
f1 (ppm)

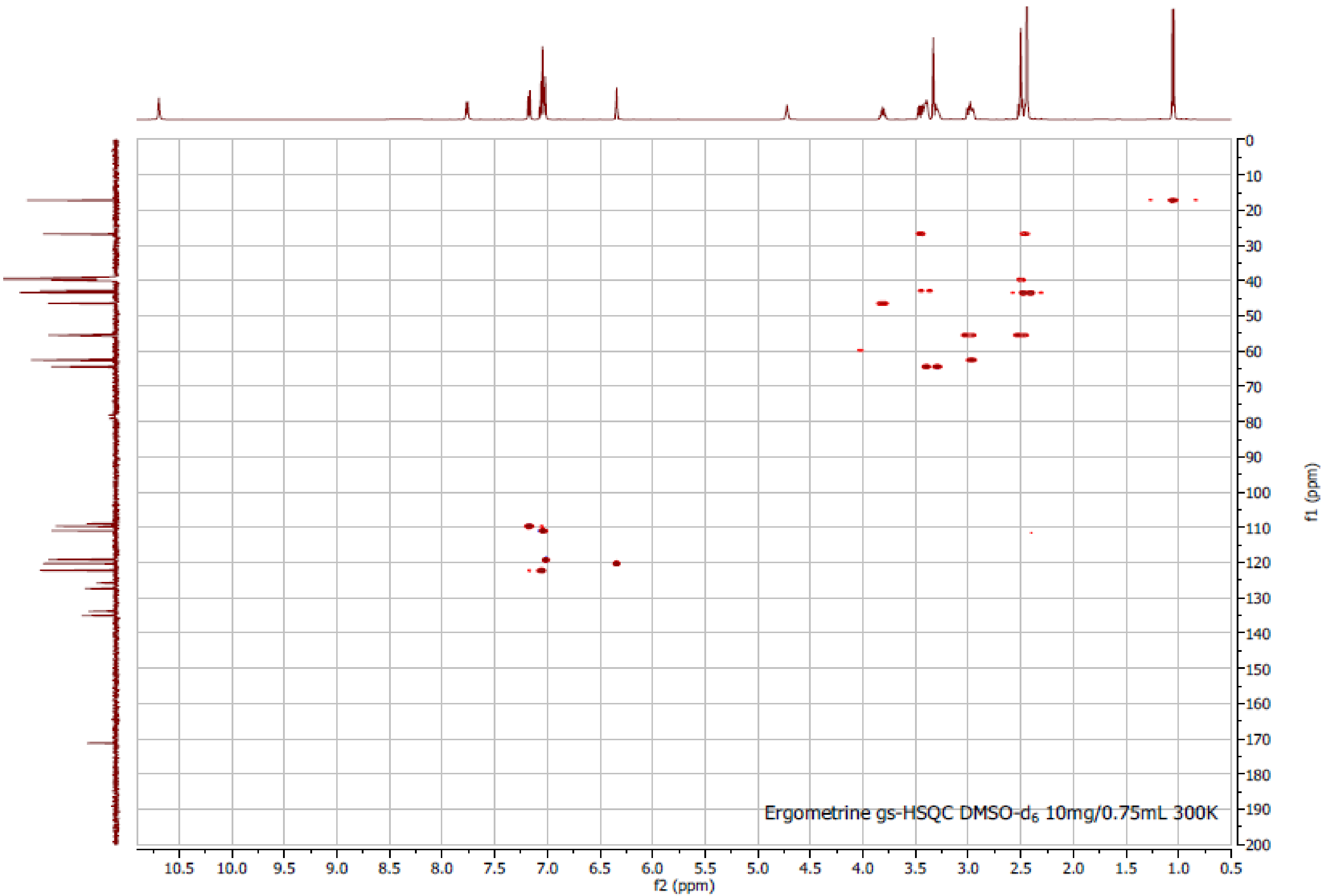
Ergometrine gs-HSQC DMSO-d6 10mg/0.75mL 300K
f2 (ppm)
f1 (ppm)

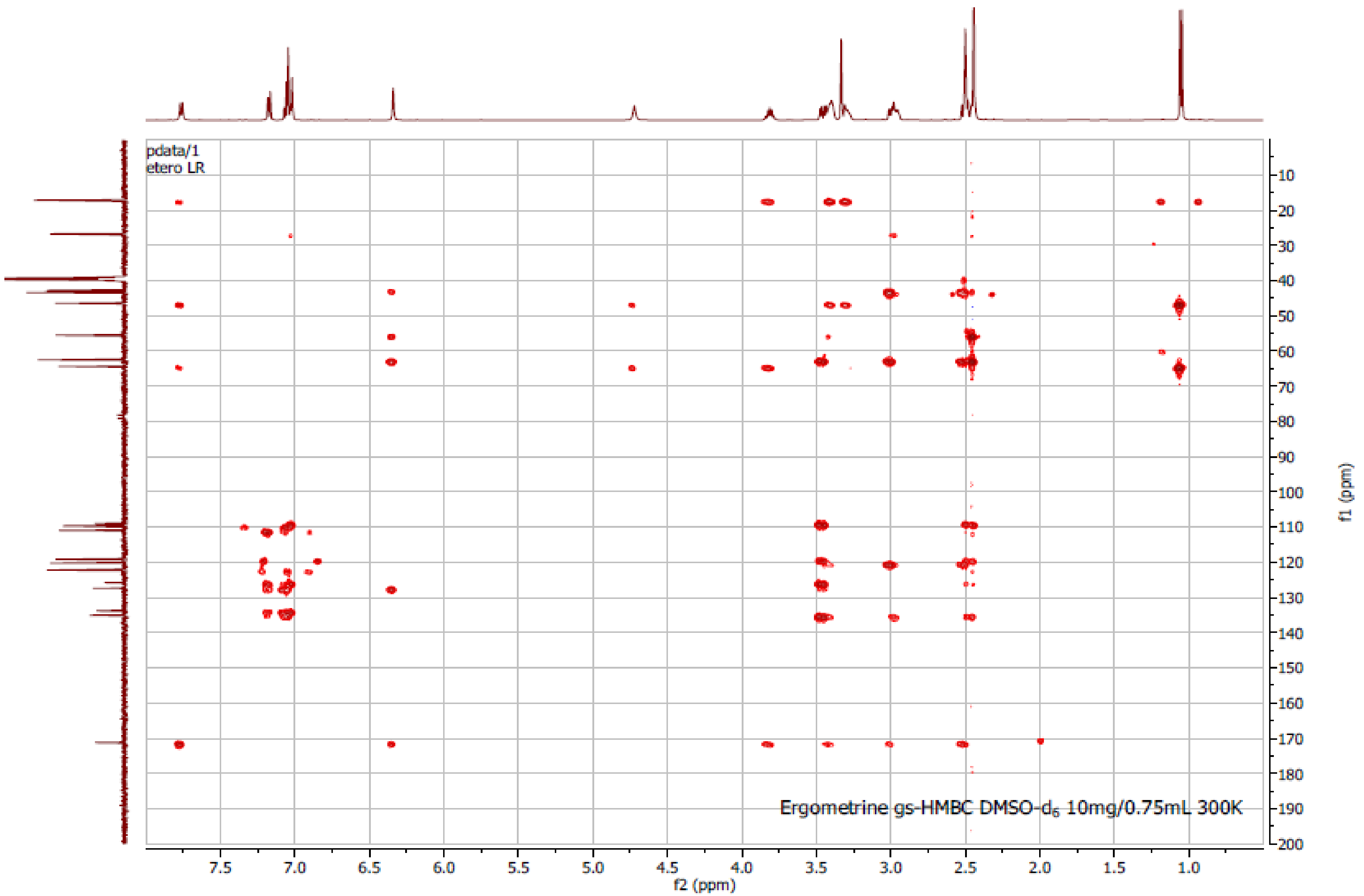
pdata/1
etero LR
Ergometrine gs-HMBC DMSO-d6 10mg/0.75mL 300K
f2 (ppm)
f1 (ppm)

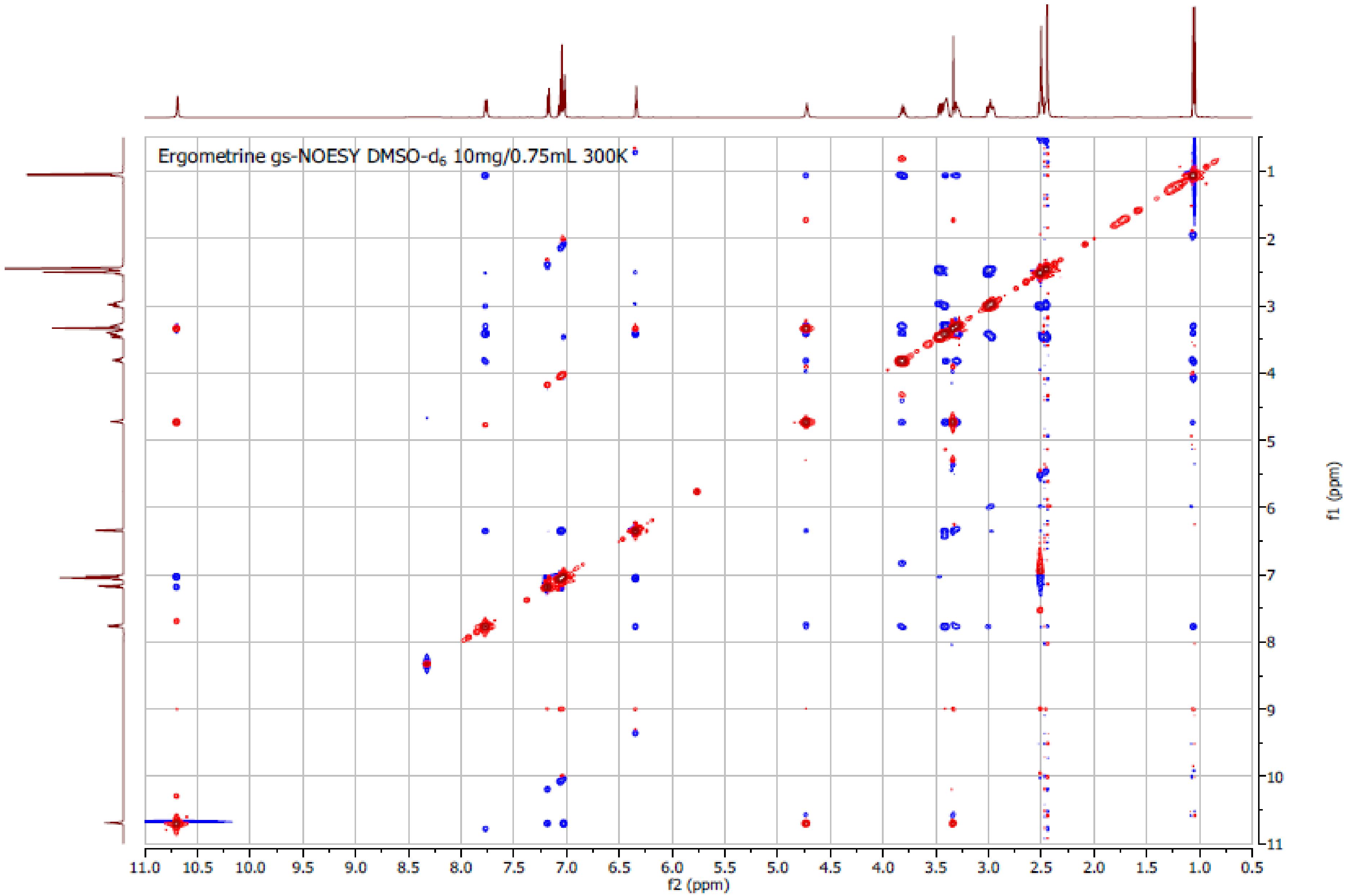
Ergometrine gs-NOESY DMSO-d_6 10mg/0.75mL 300K
f2 (ppm)
f1 (ppm)

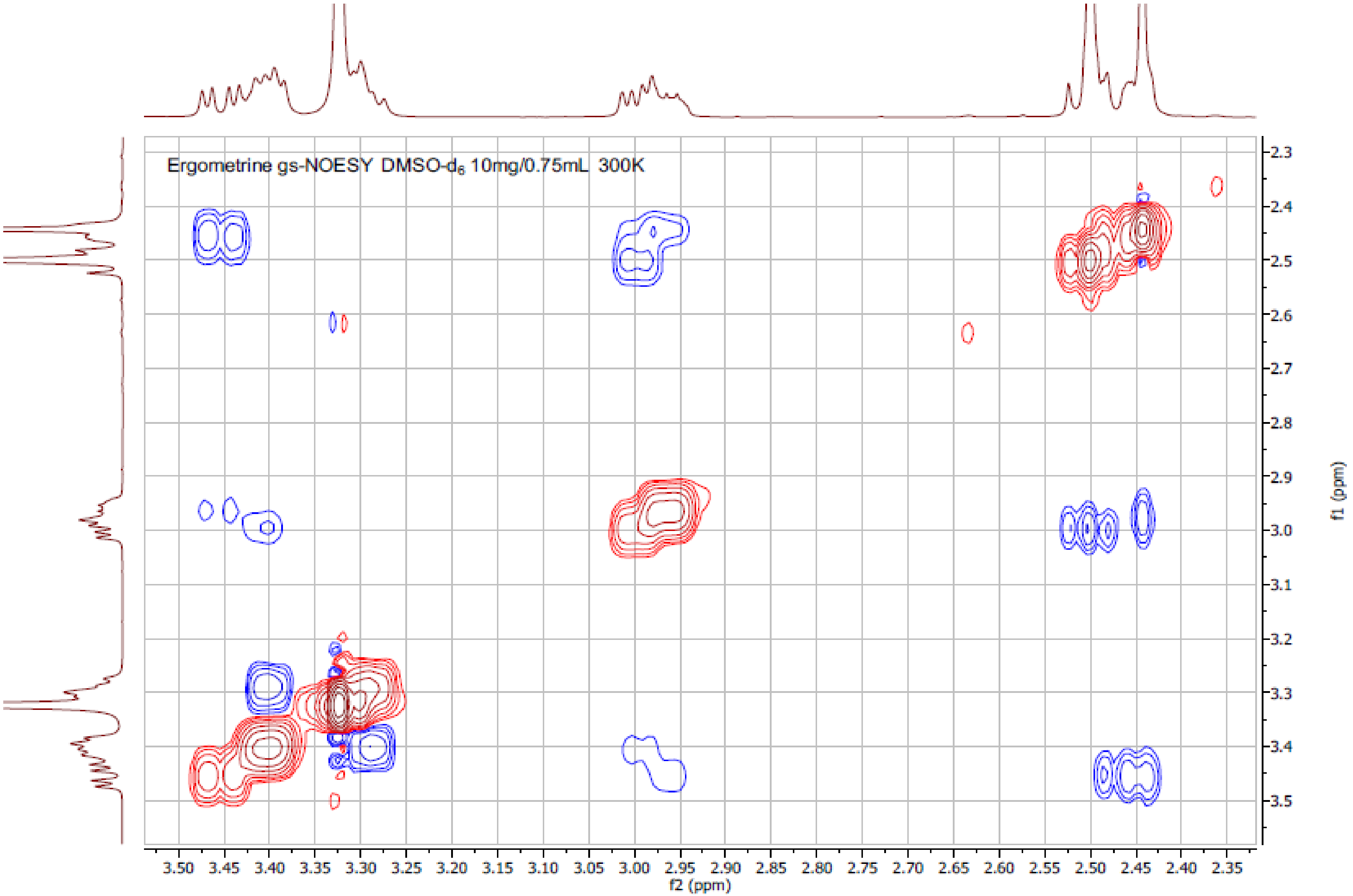

Ergometrine gs-NOESY DMSO-d6 10mg/0.75mL 300K
f2 (ppm)
f1 (ppm)

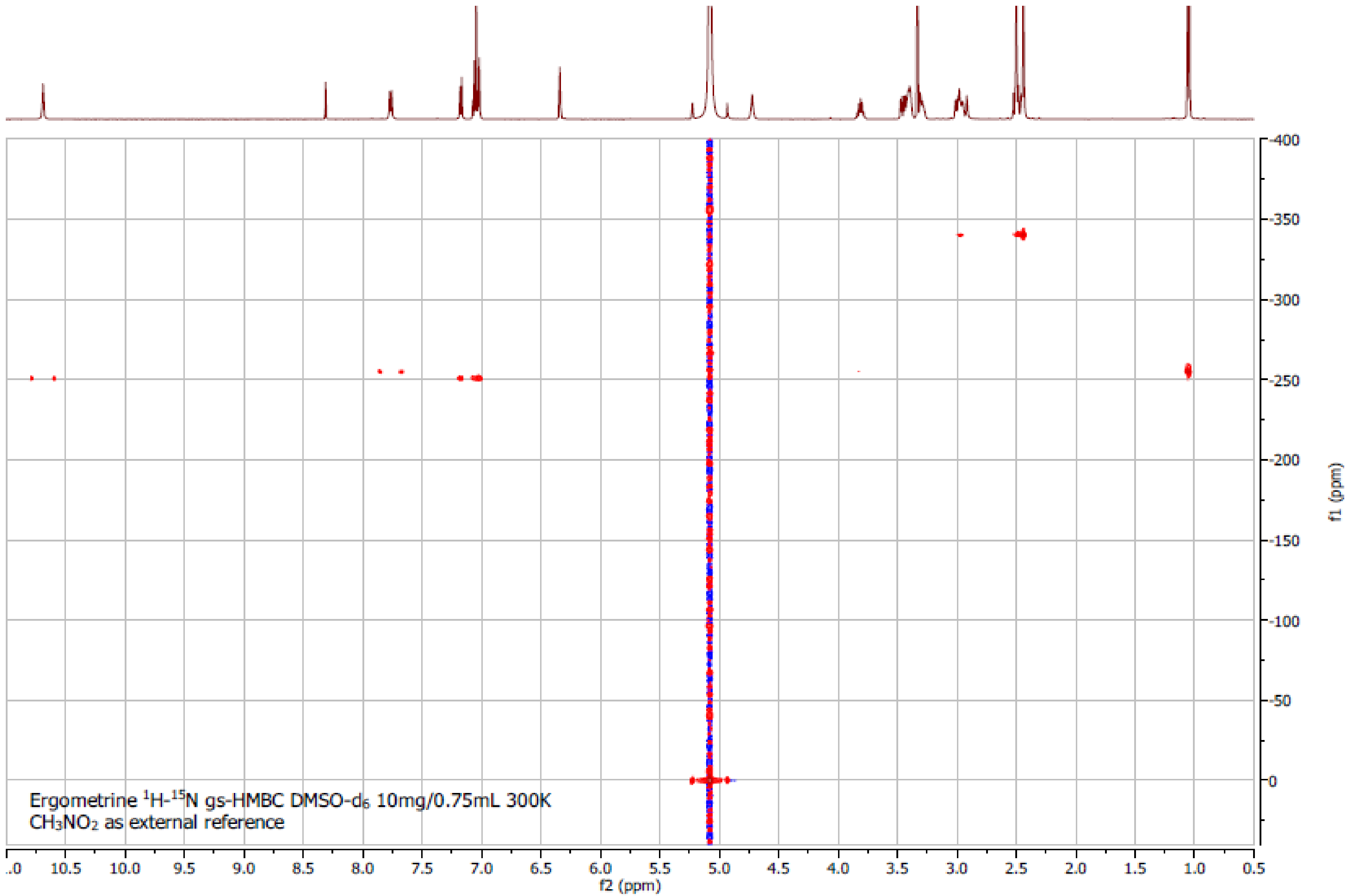

Ergometrine ¹H-¹⁵N gs-HMBC DMSO-d6 10mg/0.75mL 300K
CH3NO2 as external reference
f2 (ppm)
f1 (ppm)

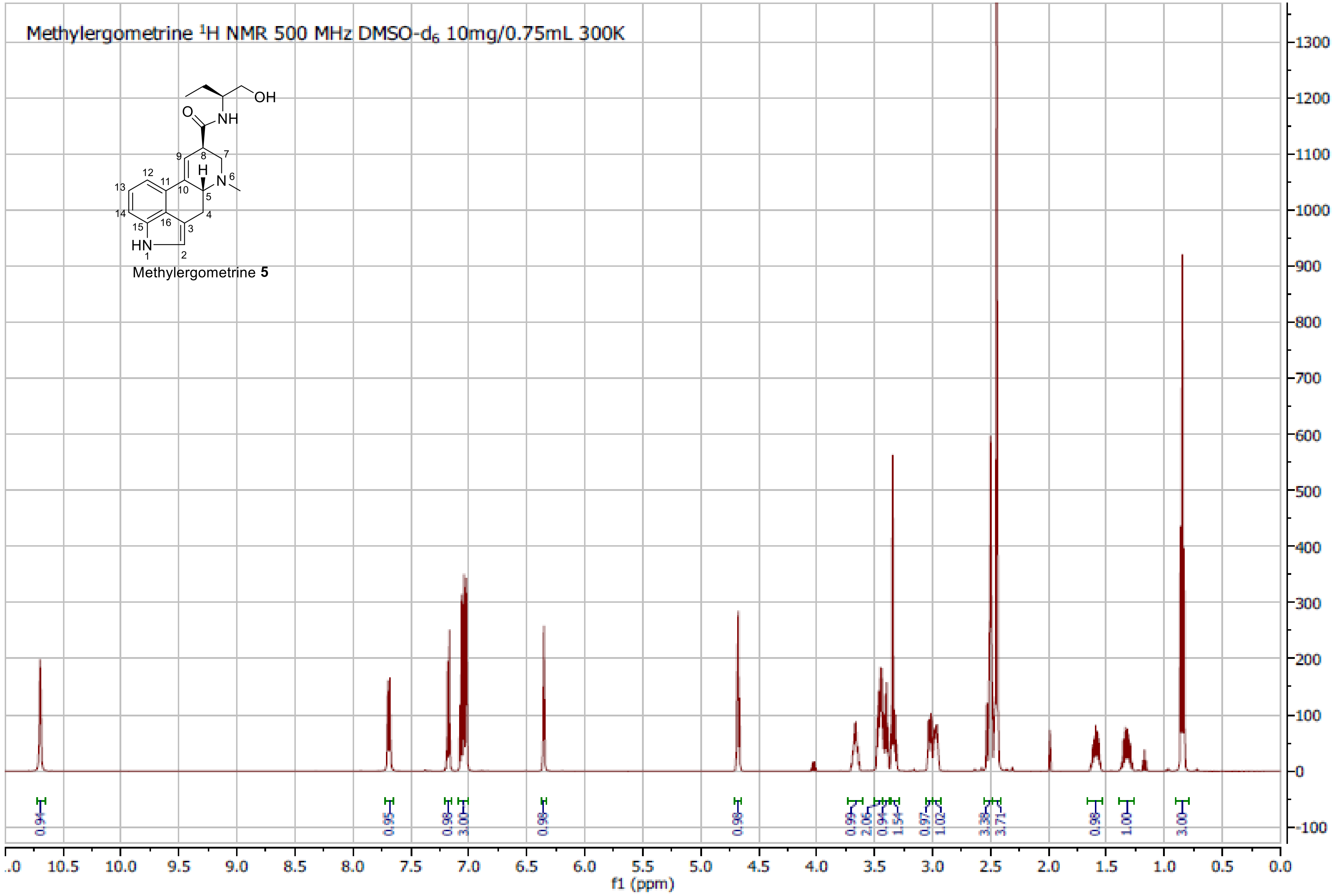
Methylergometrine ¹H NMR 500 MHz DMSO-d6 10mg/0.75mL 300K
Methylergometrine 5
f1 (ppm)

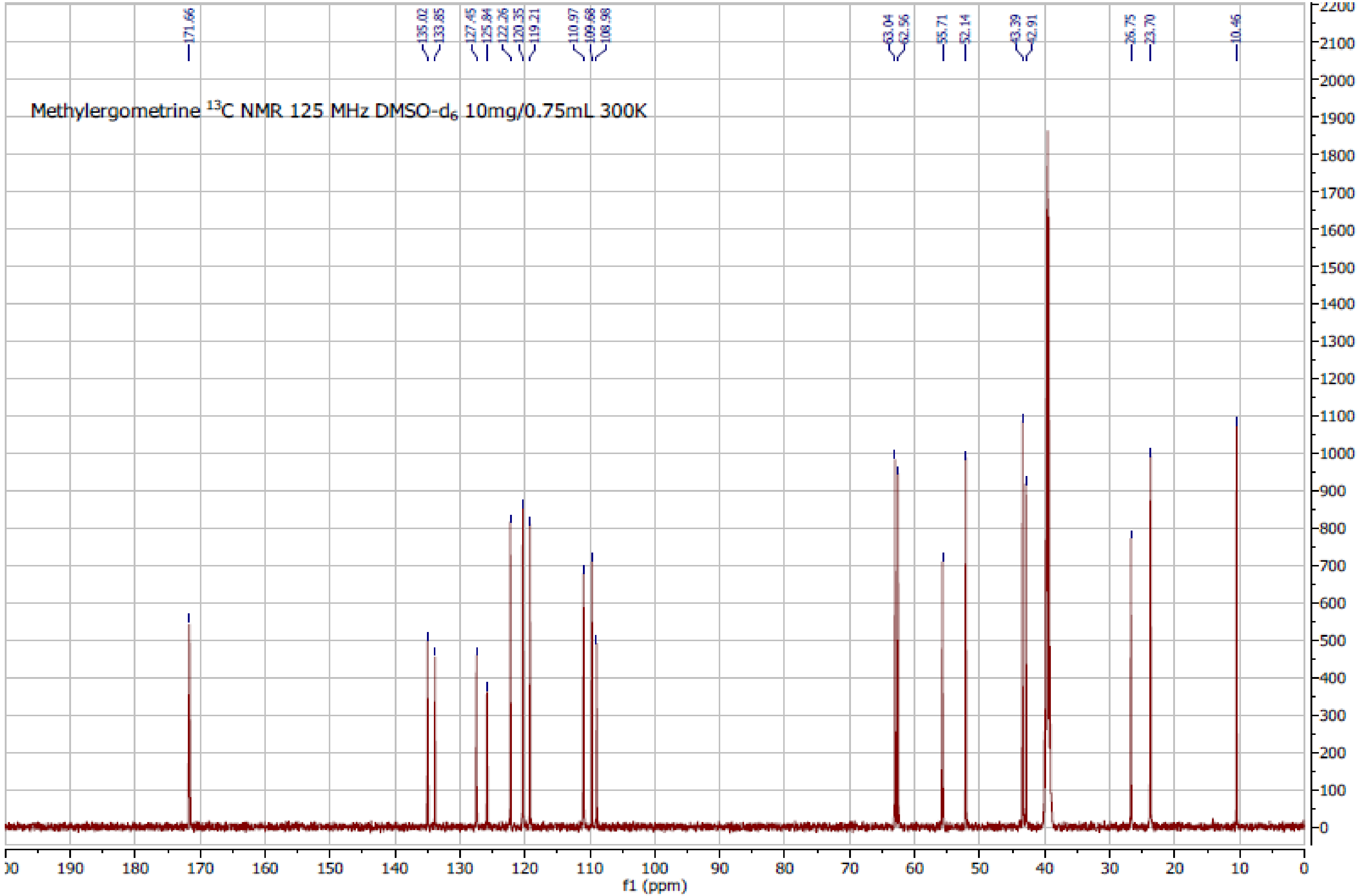

Methylergometrine ^{13}C NMR 125 MHz DMSO-d$_6$ 10mg/0.75mL 300K
171.66
135.02
133.85
127.45
125.84
122.26
120.35
119.21
110.97
109.68
108.98
63.04
62.56
55.71
52.14
43.39
42.91
26.75
23.70
10.46
f1 (ppm)

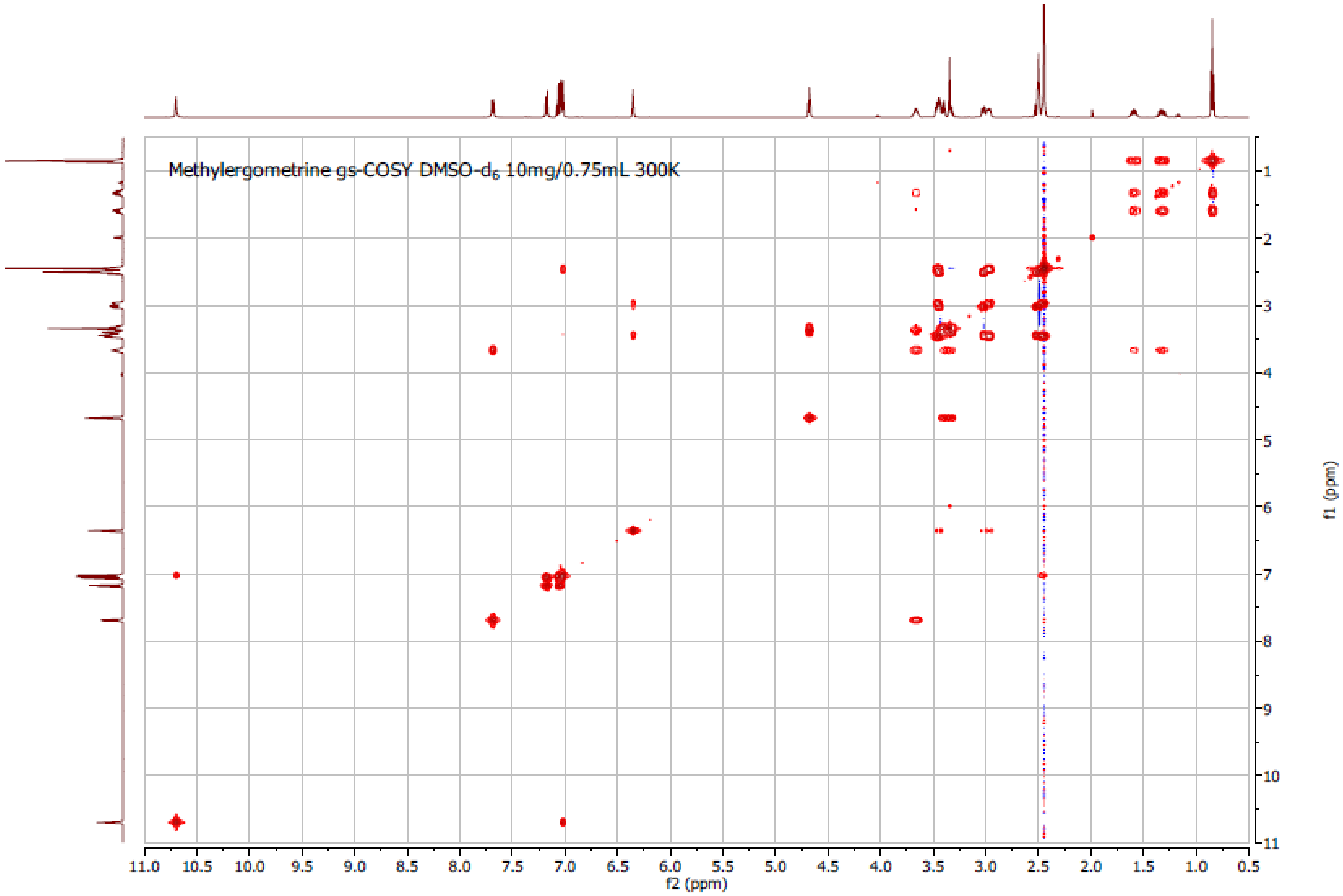
Methylergometrine gs-COSY DMSO-d6 10mg/0.75mL 300K
f2 (ppm)
f1 (ppm)
11.0
10.5
10.0
9.5
9.0
8.5
8.0
7.5
7.0
6.5
6.0
5.5
5.0
4.5
4.0
3.5
3.0
2.5
2.0
1.5
1.0
0.5
1
2
3
4
5
6
7
8
9
10
11

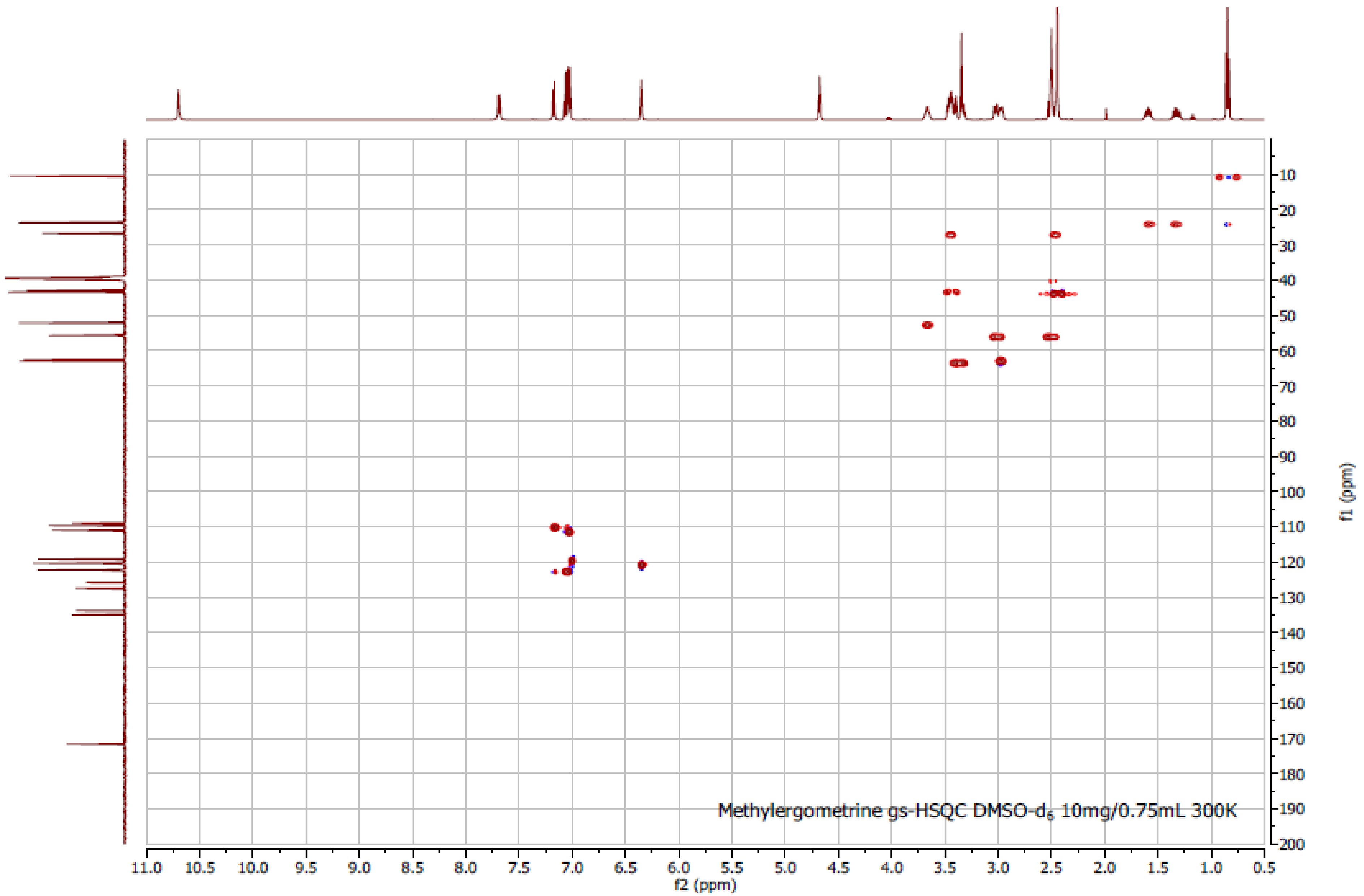
Methylergometrine gs-HSQC DMSO-d_6 10mg/0.75mL 300K
f2 (ppm)
f1 (ppm)

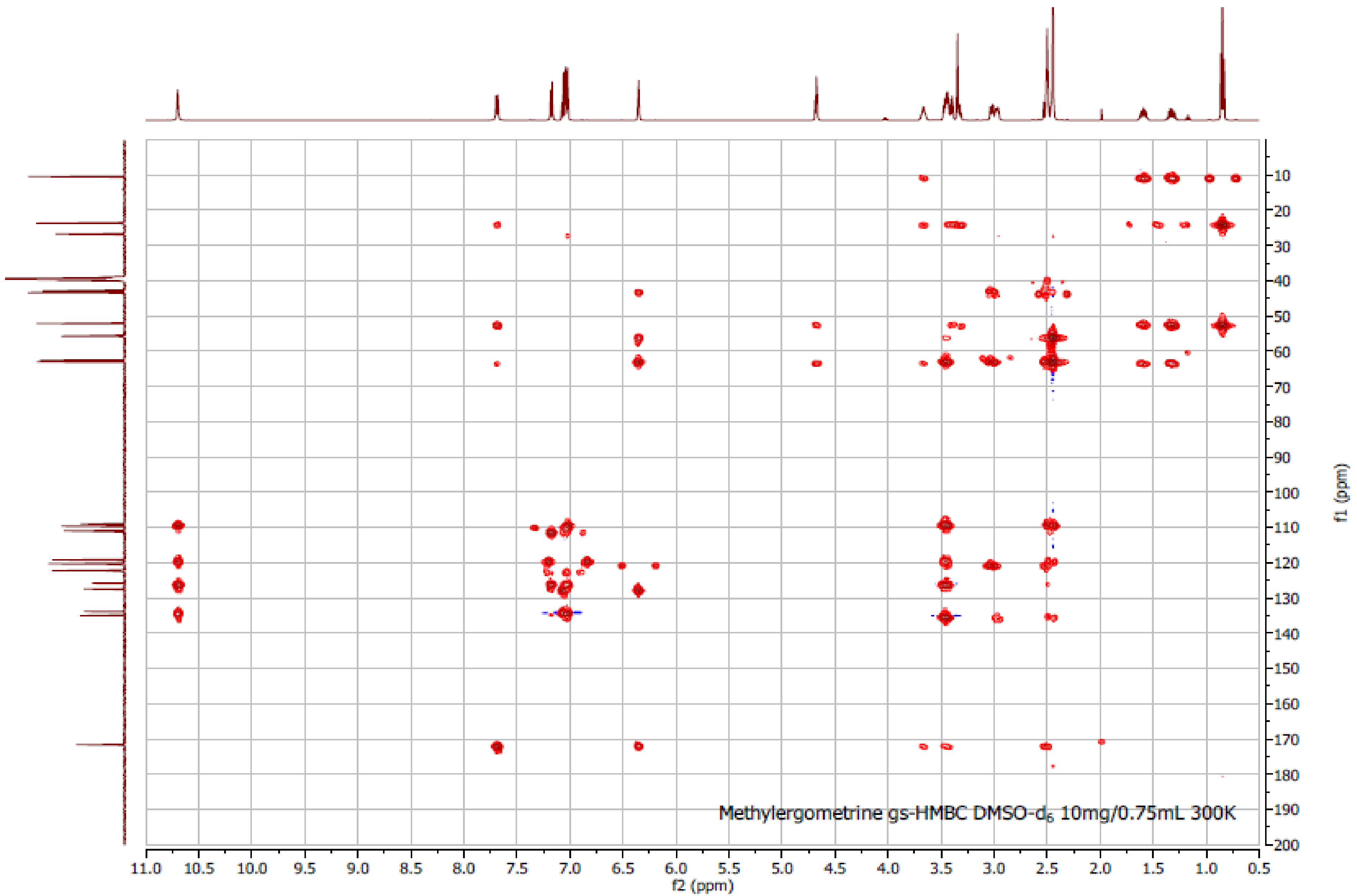

Methylergometrine gs-HMBC DMSO-d6 10mg/0.75mL 300K
f2 (ppm)
f1 (ppm)

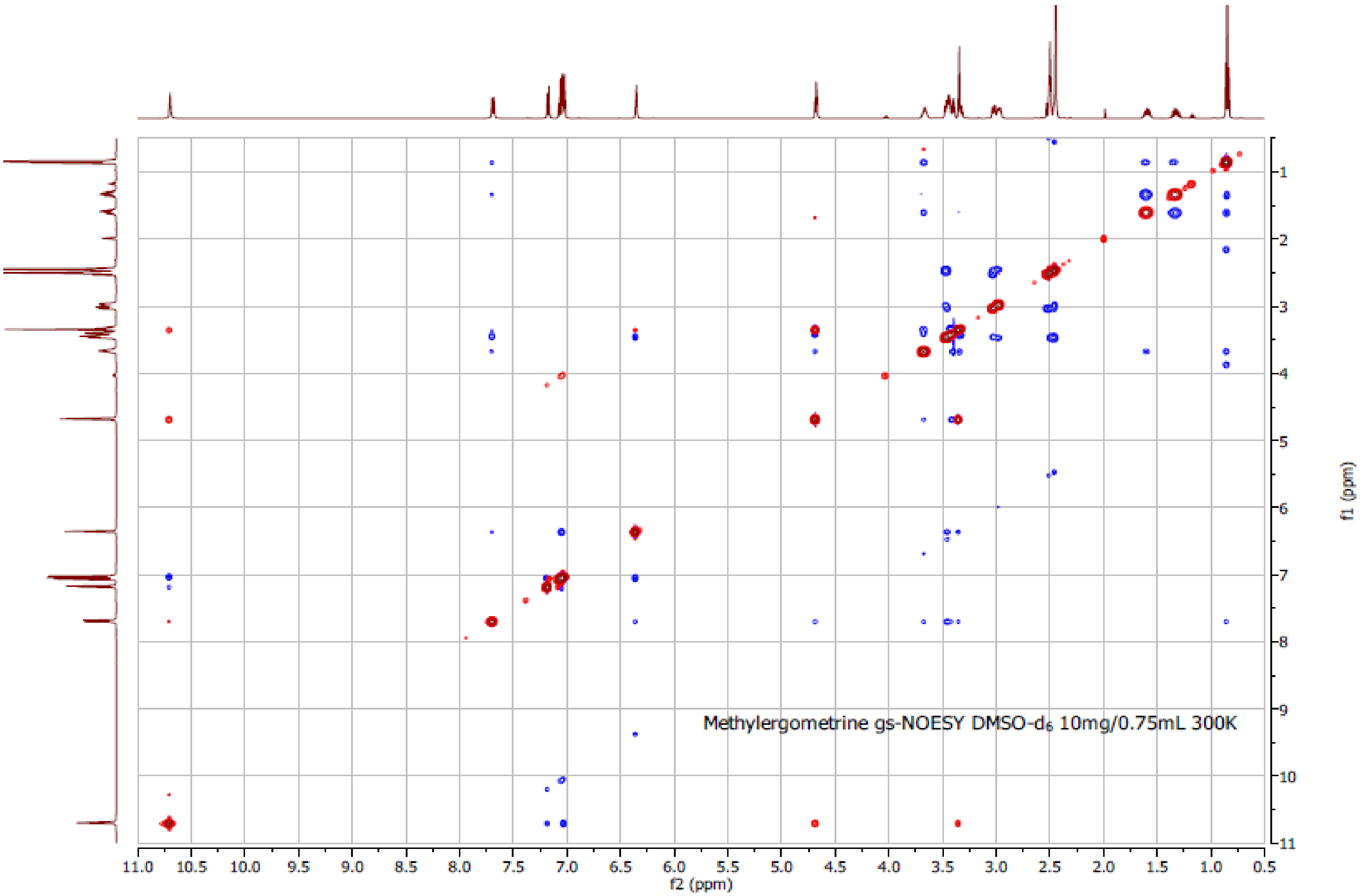

Methylergometrine gs-NOESY DMSO-d6 10mg/0.75mL 300K
f2 (ppm)
f1 (ppm)

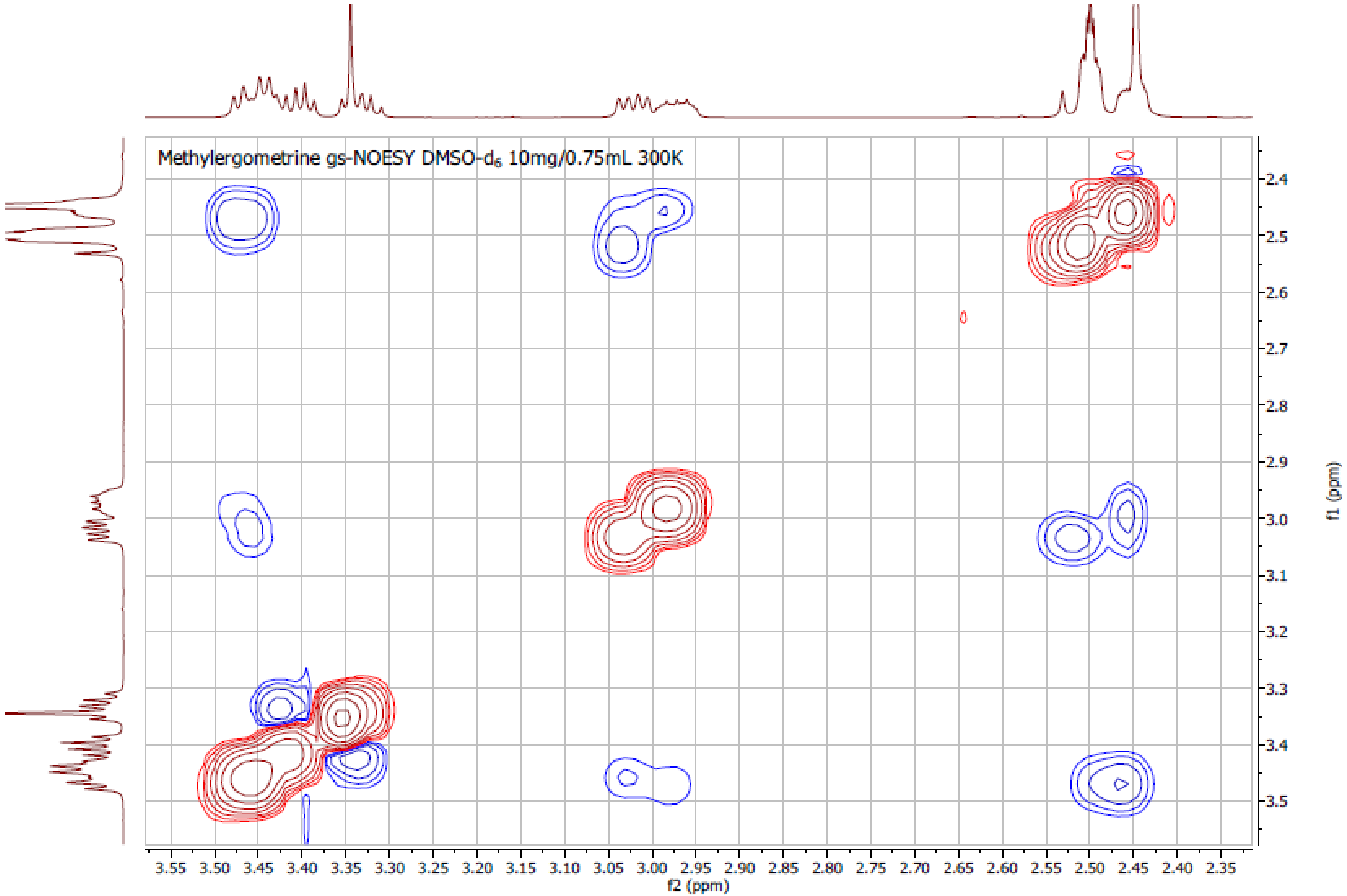

Methylergometrine gs-NOESY DMSO-d6 10mg/0.75mL 300K
3.55 3.50 3.45 3.40 3.35 3.30 3.25 3.20 3.15 3.10 3.05 3.00 2.95 2.90 2.85 2.80 2.75 2.70 2.65 2.60 2.55 2.50 2.45 2.40 2.35
f2 (ppm)
2.4 2.5 2.6 2.7 2.8 2.9 3.0 3.1 3.2 3.3 3.4 3.5
f1 (ppm)

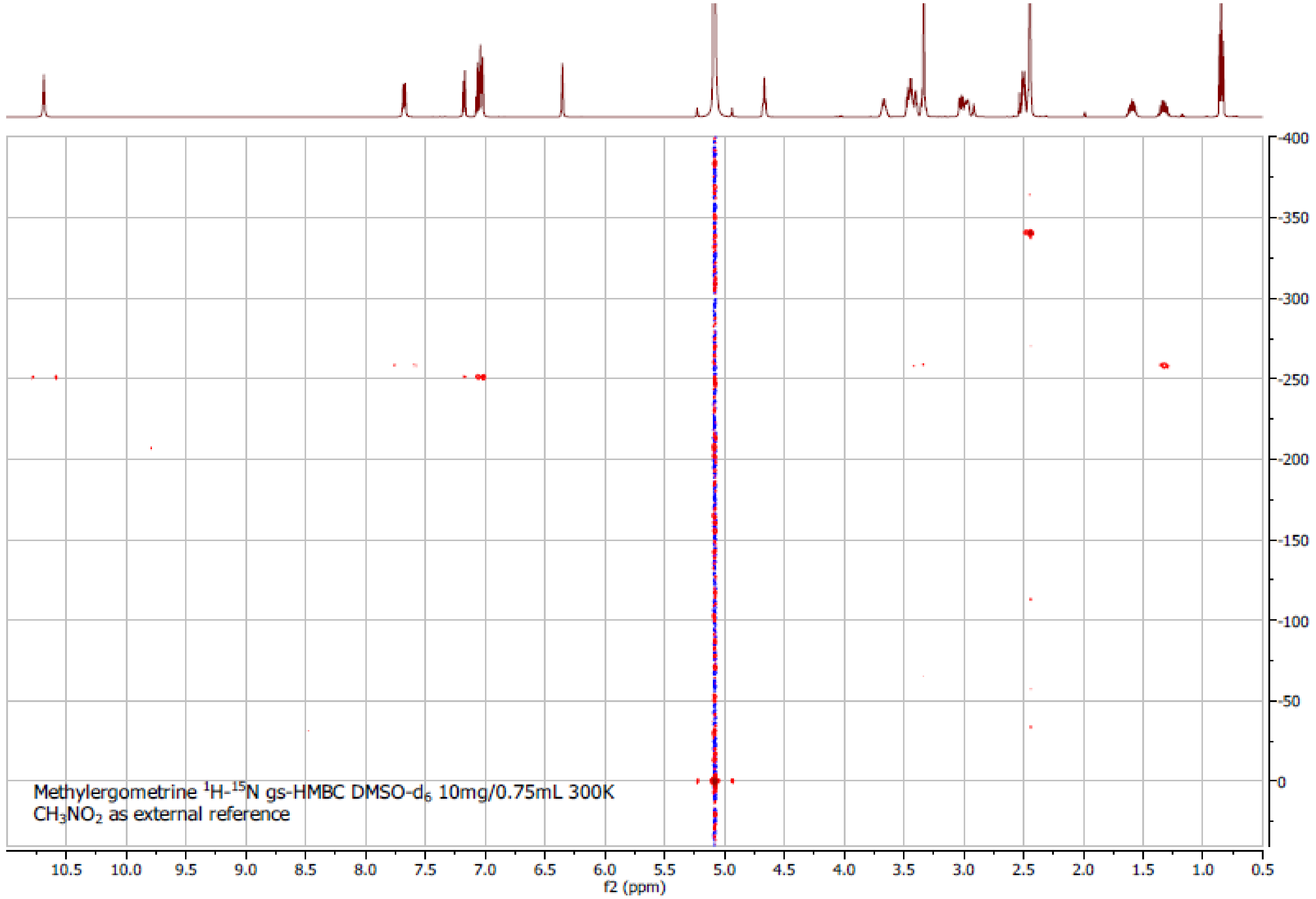

Methylergometrine ^1H-^{15}N gs-HMBC DMSO-d_6 10mg/0.75mL 300K
CH_3NO_2 as external reference
f2 (ppm)
f1 (ppm)
